# Supplementary material for: Customer tolerance in homestays: The influence of interpersonal interaction and motivation Attribution
Source: PLoS One. 2025 Oct 14;20(10):e0334636. doi: 10.1371/journal.pone.0334636 (PMC12520398; doi:10.1371/journal.pone.0334636)
Supplement: S1 — (PDF) [file pone.0334636.s001.pdf]

| | | | | | | | | | | | | | | | | | |
|---|---|---|---|---|---|---|---|---|---|---|---|---|---|---|---|---|---|
| 3 | 3 | 3 | 3 | 3 | 5 | 4 | 6 | 6 | 6 | 6 | 6 | 5 | 2 | 4 | 5 | 5 | 5 |
| 7 | 7 | 7 | 7 | 7 | 7 | 1 | 7 | 7 | 7 | 7 | 7 | 7 | 2 | 2 | 2 | 5 | 4 |
| 7 | 7 | 7 | 5 | 6 | 6 | 5 | 7 | 7 | 7 | 5 | 5 | 4 | 2 | 4 | 2 | 5 | 5 |
| 5 | 5 | 4 | 3 | 5 | 4 | 4 | 6 | 5 | 5 | 3 | 4 | 3 | 2 | 1 | 1 | 5 | 1 |
| 6 | 5 | 6 | 5 | 5 | 6 | 5 | 2 | 3 | 2 | 2 | 3 | 2 | 1 | 2 | 5 | 5 | 5 |
| 6 | 5 | 6 | 5 | 5 | 6 | 5 | 2 | 3 | 2 | 2 | 3 | 2 | 1 | 2 | 5 | 5 | 5 |
| 7 | 5 | 6 | 4 | 4 | 5 | 2 | 6 | 5 | 3 | 3 | 5 | 4 | 1 | 4 | 3 | 5 | 5 |
| 6 | 5 | 6 | 5 | 5 | 6 | 5 | 2 | 3 | 2 | 2 | 3 | 2 | 1 | 2 | 5 | 5 | 5 |
| 6 | 5 | 6 | 5 | 5 | 6 | 5 | 2 | 3 | 2 | 3 | 2 | 3 | 1 | 3 | 5 | 5 | 5 |
| 6 | 6 | 6 | 6 | 4 | 5 | 4 | 6 | 5 | 6 | 5 | 5 | 4 | 2 | 3 | 3 | 5 | 2 |
| 5 | 6 | 7 | 7 | 5 | 6 | 5 | 5 | 5 | 6 | 6 | 6 | 5 | 2 | 2 | 2 | 5 | 3 |
| 6 | 5 | 7 | 7 | 6 | 7 | 6 | 3 | 1 | 2 | 6 | 7 | 6 | 2 | 2 | 6 | 5 | 5 |
| 7 | 7 | 6 | 7 | 6 | 7 | 7 | 2 | 1 | 3 | 5 | 7 | 6 | 2 | 3 | 3 | 5 | 4 |
| 6 | 5 | 6 | 5 | 6 | 5 | 6 | 2 | 3 | 2 | 2 | 3 | 2 | 2 | 3 | 5 | 5 | 5 |
| 7 | 6 | 7 | 7 | 7 | 6 | 7 | 2 | 1 | 3 | 5 | 6 | 7 | 2 | 3 | 2 | 5 | 4 |
| 7 | 7 | 6 | 7 | 7 | 7 | 6 | 6 | 5 | 5 | 2 | 6 | 7 | 2 | 3 | 2 | 5 | 5 |
| 7 | 7 | 6 | 7 | 7 | 7 | 6 | 6 | 5 | 5 | 2 | 6 | 7 | 2 | 3 | 2 | 5 | 5 |
| 7 | 7 | 6 | 7 | 7 | 7 | 6 | 6 | 5 | 5 | 3 | 7 | 6 | 2 | 3 | 2 | 5 | 5 |
| 7 | 7 | 6 | 7 | 7 | 7 | 6 | 6 | 5 | 5 | 3 | 7 | 6 | 2 | 3 | 2 | 5 | 5 |
| 7 | 6 | 7 | 7 | 7 | 6 | 7 | 3 | 1 | 2 | 5 | 7 | 7 | 2 | 2 | 2 | 5 | 4 |
| 7 | 7 | 7 | 7 | 7 | 7 | 1 | 7 | 7 | 7 | 7 | 7 | 7 | 2 | 2 | 2 | 5 | 4 |
| 6 | 5 | 6 | 5 | 5 | 6 | 5 | 2 | 3 | 2 | 2 | 3 | 2 | 2 | 3 | 5 | 5 | 5 |
| 7 | 6 | 7 | 6 | 7 | 6 | 7 | 6 | 1 | 7 | 7 | 6 | 7 | 2 | 2 | 5 | 5 | 4 |
| 7 | 7 | 4 | 7 | 7 | 6 | 4 | 4 | 4 | 5 | 2 | 5 | 4 | 1 | 4 | 9 | 2 | 1 |
| 7 | 5 | 6 | 5 | 6 | 7 | 3 | 4 | 3 | 5 | 6 | 5 | 2 | 1 | 2 | 2 | 6 | 5 |
| 6 | 5 | 6 | 5 | 6 | 5 | 6 | 3 | 2 | 3 | 2 | 3 | 2 | 1 | 3 | 5 | 5 | 5 |
| 5 | 6 | 5 | 6 | 7 | 6 | 2 | 4 | 2 | 6 | 5 | 6 | 3 | 1 | 3 | 6 | 5 | 5 |
| 6 | 5 | 6 | 6 | 5 | 6 | 6 | 3 | 3 | 4 | 5 | 6 | 5 | 2 | 2 | 5 | 4 | 5 |
| 6 | 5 | 6 | 5 | 6 | 7 | 6 | 4 | 3 | 4 | 5 | 6 | 6 | 2 | 3 | 5 | 4 | 5 |
| 7 | 7 | 6 | 6 | 5 | 6 | 5 | 5 | 3 | 5 | 5 | 6 | 5 | 2 | 2 | 5 | 5 | 5 |
| 6 | 6 | 7 | 5 | 6 | 6 | 5 | 6 | 4 | 6 | 3 | 6 | 4 | 1 | 2 | 2 | 5 | 4 |
| 6 | 7 | 6 | 7 | 7 | 6 | 7 | 6 | 4 | 5 | 5 | 6 | 5 | 2 | 2 | 5 | 5 | 5 |
| 7 | 6 | 7 | 6 | 6 | 7 | 6 | 7 | 5 | 6 | 6 | 5 | 6 | 2 | 2 | 5 | 5 | 4 |
| 6 | 5 | 6 | 7 | 6 | 5 | 6 | 6 | 7 | 6 | 5 | 7 | 6 | 1 | 3 | 5 | 5 | 5 |
| 7 | 6 | 7 | 6 | 6 | 7 | 7 | 6 | 5 | 7 | 3 | 2 | 5 | 2 | 2 | 3 | 5 | 4 |
| 5 | 7 | 6 | 6 | 6 | 7 | 5 | 2 | 1 | 2 | 5 | 6 | 7 | 1 | 2 | 5 | 5 | 5 |
| 6 | 7 | 7 | 6 | 7 | 7 | 6 | 2 | 1 | 3 | 5 | 6 | 7 | 2 | 2 | 2 | 5 | 4 |
| 6 | 6 | 5 | 6 | 5 | 6 | 4 | 6 | 5 | 7 | 3 | 5 | 4 | 1 | 2 | 5 | 4 | 4 |
| 6 | 6 | 6 | 7 | 7 | 7 | 6 | 2 | 1 | 3 | 5 | 6 | 7 | 1 | 2 | 5 | 5 | 5 |
| 6 | 7 | 5 | 6 | 7 | 6 | 7 | 2 | 1 | 3 | 5 | 6 | 7 | 2 | 2 | 5 | 5 | 4 |
| 7 | 6 | 6 | 5 | 5 | 5 | 5 | 7 | 7 | 7 | 2 | 5 | 4 | 2 | 4 | 5 | 4 | 4 |
| 7 | 7 | 6 | 6 | 5 | 5 | 4 | 5 | 6 | 5 | 2 | 5 | 4 | 1 | 2 | 2 | 5 | 5 |
| 6 | 6 | 7 | 6 | 5 | 6 | 7 | 5 | 6 | 7 | 6 | 7 | 6 | 2 | 2 | 5 | 5 | 5 |
| 7 | 6 | 6 | 5 | 5 | 6 | 4 | 4 | 2 | 3 | 4 | 5 | 5 | 2 | 2 | 5 | 5 | 5 |

| | | | | | | | | | | | | | | | | | |
|---|---|---|---|---|---|---|---|---|---|---|---|---|---|---|---|---|---|
| 7 | 4 | 5 | 6 | 6 | 7 | 4 | 6 | 5 | 6 | 5 | 6 | 5 | 2 | 2 | 6 | 5 | 5 |
| 6 | 6 | 5 | 7 | 5 | 5 | 3 | 6 | 6 | 6 | 4 | 6 | 3 | 2 | 1 | 1 | 3 | 1 |
| 7 | 6 | 6 | 7 | 6 | 7 | 7 | 6 | 7 | 5 | 4 | 5 | 5 | 2 | 2 | 5 | 4 | 3 |
| 7 | 5 | 6 | 7 | 7 | 5 | 6 | 7 | 5 | 7 | 3 | 5 | 5 | 2 | 3 | 4 | 5 | 5 |
| 7 | 7 | 5 | 6 | 5 | 6 | 4 | 6 | 5 | 5 | 6 | 6 | 6 | 2 | 3 | 3 | 4 | 2 |
| 6 | 7 | 5 | 7 | 6 | 7 | 5 | 7 | 5 | 7 | 2 | 5 | 6 | 2 | 3 | 5 | 6 | 5 |
| 6 | 6 | 7 | 6 | 6 | 6 | 7 | 7 | 6 | 7 | 3 | 6 | 5 | 2 | 3 | 5 | 5 | 5 |
| 7 | 5 | 6 | 6 | 6 | 6 | 6 | 7 | 6 | 5 | 5 | 5 | 5 | 2 | 2 | 2 | 5 | 5 |
| 2 | 6 | 6 | 1 | 4 | 5 | 5 | 6 | 4 | 5 | 2 | 6 | 4 | 2 | 1 | 1 | 5 | 1 |
| 6 | 7 | 6 | 7 | 6 | 6 | 6 | 6 | 6 | 6 | 6 | 6 | 6 | 2 | 2 | 3 | 5 | 5 |
| 6 | 6 | 6 | 6 | 4 | 6 | 4 | 7 | 7 | 7 | 3 | 4 | 4 | 2 | 3 | 2 | 5 | 5 |
| 6 | 7 | 5 | 6 | 6 | 5 | 7 | 5 | 3 | 2 | 5 | 6 | 7 | 2 | 2 | 1 | 6 | 4 |
| 6 | 3 | 5 | 6 | 4 | 5 | 3 | 6 | 6 | 7 | 2 | 5 | 3 | 2 | 2 | 5 | 5 | 3 |
| 6 | 3 | 3 | 3 | 3 | 3 | 2 | 6 | 6 | 5 | 3 | 5 | 4 | 2 | 2 | 5 | 5 | 4 |
| 7 | 6 | 6 | 7 | 7 | 7 | 7 | 6 | 4 | 7 | 4 | 4 | 4 | 2 | 3 | 2 | 5 | 5 |
| 6 | 7 | 6 | 5 | 6 | 6 | 7 | 5 | 3 | 3 | 5 | 6 | 6 | 2 | 3 | 2 | 6 | 5 |
| 6 | 7 | 6 | 7 | 6 | 5 | 7 | 4 | 3 | 4 | 5 | 5 | 6 | 2 | 2 | 1 | 5 | 2 |
| 7 | 6 | 6 | 6 | 6 | 6 | 6 | 5 | 3 | 5 | 3 | 6 | 5 | 1 | 2 | 5 | 5 | 5 |
| 5 | 5 | 6 | 7 | 6 | 7 | 7 | 7 | 6 | 6 | 3 | 2 | 4 | 1 | 3 | 3 | 5 | 3 |
| 7 | 6 | 6 | 7 | 7 | 7 | 7 | 7 | 7 | 7 | 1 | 7 | 4 | 2 | 3 | 9 | 3 | 5 |
| 6 | 5 | 6 | 7 | 6 | 7 | 5 | 6 | 5 | 6 | 2 | 4 | 3 | 2 | 3 | 3 | 5 | 5 |
| 6 | 6 | 7 | 6 | 5 | 5 | 4 | 7 | 7 | 7 | 2 | 5 | 3 | 2 | 3 | 5 | 4 | 5 |
| 7 | 7 | 7 | 3 | 7 | 7 | 4 | 7 | 6 | 6 | 6 | 7 | 6 | 2 | 4 | 4 | 5 | 5 |
| 7 | 7 | 7 | 7 | 7 | 7 | 7 | 7 | 6 | 7 | 6 | 7 | 6 | 2 | 3 | 5 | 5 | 5 |
| 6 | 6 | 5 | 6 | 6 | 6 | 4 | 6 | 7 | 6 | 3 | 4 | 3 | 2 | 2 | 5 | 5 | 4 |
| 6 | 6 | 6 | 3 | 4 | 3 | 3 | 3 | 5 | 5 | 5 | 4 | 3 | 1 | 3 | 3 | 5 | 5 |
| 6 | 7 | 6 | 6 | 6 | 7 | 6 | 6 | 6 | 6 | 1 | 5 | 2 | 2 | 2 | 2 | 5 | 5 |
| 7 | 7 | 6 | 6 | 7 | 5 | 5 | 6 | 3 | 5 | 1 | 6 | 3 | 1 | 2 | 3 | 5 | 4 |
| 6 | 7 | 6 | 6 | 5 | 6 | 5 | 7 | 3 | 5 | 5 | 5 | 6 | 1 | 3 | 5 | 5 | 5 |
| 6 | 5 | 6 | 5 | 5 | 6 | 5 | 2 | 3 | 2 | 3 | 2 | 3 | 1 | 2 | 5 | 5 | 5 |
| 6 | 7 | 6 | 6 | 6 | 7 | 6 | 7 | 6 | 5 | 2 | 1 | 4 | 2 | 2 | 5 | 5 | 4 |
| 6 | 6 | 6 | 7 | 6 | 6 | 7 | 7 | 6 | 6 | 4 | 5 | 5 | 2 | 3 | 5 | 5 | 4 |
| 7 | 6 | 7 | 6 | 7 | 6 | 7 | 6 | 7 | 6 | 2 | 3 | 4 | 2 | 3 | 2 | 5 | 4 |
| 6 | 6 | 6 | 6 | 5 | 5 | 6 | 6 | 6 | 5 | 2 | 3 | 4 | 2 | 2 | 3 | 5 | 3 |
| 7 | 7 | 7 | 5 | 6 | 5 | 5 | 3 | 3 | 3 | 6 | 6 | 6 | 1 | 1 | 1 | 4 | 3 |
| 3 | 6 | 2 | 2 | 3 | 2 | 2 | 6 | 5 | 5 | 6 | 6 | 5 | 1 | 1 | 1 | 5 | 1 |
| 6 | 6 | 5 | 6 | 5 | 5 | 4 | 5 | 3 | 6 | 5 | 6 | 7 | 2 | 2 | 1 | 6 | 1 |
| 6 | 6 | 6 | 5 | 5 | 5 | 4 | 6 | 6 | 6 | 3 | 5 | 4 | 1 | 2 | 1 | 6 | 1 |
| 6 | 6 | 6 | 5 | 6 | 6 | 6 | 6 | 6 | 6 | 3 | 5 | 5 | 1 | 5 | 5 | 2 | 3 |
| 7 | 7 | 7 | 6 | 6 | 6 | 5 | 6 | 6 | 5 | 5 | 5 | 4 | 1 | 2 | 1 | 6 | 1 |
| 5 | 6 | 5 | 5 | 5 | 5 | 5 | 6 | 5 | 6 | 3 | 3 | 3 | 1 | 1 | 1 | 5 | 4 |
| 6 | 5 | 6 | 5 | 5 | 6 | 5 | 2 | 3 | 2 | 2 | 3 | 2 | 2 | 2 | 5 | 5 | 5 |
| 7 | 7 | 6 | 7 | 6 | 7 | 6 | 3 | 3 | 5 | 3 | 6 | 6 | 1 | 2 | 5 | 5 | 4 |
| 6 | 6 | 5 | 5 | 5 | 6 | 6 | 3 | 2 | 3 | 6 | 5 | 5 | 1 | 3 | 5 | 4 | 4 |

| | | | | | | | | | | | | | | | | | |
|---|---|---|---|---|---|---|---|---|---|---|---|---|---|---|---|---|---|
| 6 | 5 | 6 | 5 | 5 | 6 | 5 | 2 | 3 | 2 | 2 | 3 | 2 | 2 | 2 | 5 | 5 | 5 |
| 3 | 5 | 4 | 2 | 3 | 5 | 4 | 7 | 7 | 6 | 5 | 3 | 3 | 2 | 1 | 1 | 5 | 1 |
| 7 | 6 | 6 | 7 | 6 | 7 | 4 | 6 | 6 | 6 | 2 | 4 | 3 | 1 | 4 | 5 | 5 | 5 |
| 6 | 5 | 6 | 5 | 5 | 6 | 5 | 2 | 3 | 2 | 2 | 3 | 2 | 1 | 2 | 5 | 5 | 5 |
| 6 | 6 | 6 | 5 | 6 | 6 | 6 | 5 | 5 | 5 | 4 | 6 | 5 | 1 | 2 | 5 | 5 | 5 |
| 5 | 3 | 3 | 3 | 3 | 3 | 3 | 5 | 5 | 5 | 2 | 5 | 2 | 2 | 2 | 3 | 5 | 4 |
| 5 | 5 | 5 | 6 | 5 | 5 | 5 | 6 | 5 | 5 | 5 | 5 | 5 | 2 | 2 | 2 | 5 | 3 |
| 7 | 7 | 7 | 7 | 7 | 7 | 1 | 7 | 7 | 7 | 7 | 7 | 7 | 1 | 2 | 2 | 5 | 4 |
| 6 | 5 | 5 | 3 | 5 | 5 | 3 | 6 | 5 | 5 | 5 | 5 | 5 | 2 | 2 | 1 | 6 | 2 |
| 3 | 3 | 3 | 2 | 2 | 3 | 2 | 6 | 6 | 6 | 3 | 5 | 2 | 2 | 2 | 1 | 6 | 3 |
| 6 | 7 | 6 | 5 | 6 | 6 | 5 | 4 | 3 | 2 | 3 | 5 | 4 | 2 | 2 | 5 | 5 | 4 |
| 7 | 7 | 6 | 7 | 6 | 7 | 5 | 5 | 5 | 5 | 3 | 5 | 4 | 2 | 3 | 2 | 5 | 5 |
| 7 | 6 | 7 | 7 | 7 | 7 | 6 | 3 | 2 | 2 | 6 | 7 | 7 | 1 | 3 | 5 | 5 | 5 |
| 7 | 6 | 7 | 6 | 7 | 5 | 6 | 1 | 1 | 1 | 3 | 5 | 2 | 2 | 2 | 3 | 5 | 5 |
| 7 | 6 | 7 | 7 | 7 | 6 | 7 | 7 | 7 | 7 | 1 | 5 | 5 | 1 | 3 | 3 | 5 | 5 |
| 6 | 6 | 6 | 6 | 6 | 5 | 6 | 5 | 5 | 3 | 3 | 5 | 5 | 2 | 4 | 7 | 3 | 5 |
| 5 | 6 | 6 | 6 | 6 | 7 | 4 | 4 | 3 | 3 | 3 | 6 | 4 | 1 | 2 | 5 | 5 | 5 |
| 6 | 5 | 6 | 6 | 6 | 7 | 6 | 7 | 7 | 6 | 3 | 5 | 5 | 1 | 4 | 8 | 4 | 3 |
| 7 | 6 | 5 | 6 | 6 | 7 | 5 | 5 | 2 | 3 | 2 | 5 | 3 | 2 | 2 | 5 | 5 | 5 |
| 7 | 6 | 7 | 7 | 7 | 6 | 6 | 3 | 1 | 3 | 6 | 7 | 6 | 1 | 3 | 5 | 5 | 5 |
| 7 | 6 | 4 | 5 | 6 | 6 | 4 | 4 | 3 | 5 | 3 | 5 | 4 | 1 | 3 | 4 | 5 | 4 |
| 7 | 6 | 7 | 7 | 7 | 6 | 6 | 6 | 2 | 3 | 6 | 7 | 6 | 1 | 3 | 2 | 5 | 5 |
| 6 | 7 | 7 | 6 | 6 | 5 | 6 | 3 | 5 | 6 | 2 | 6 | 5 | 1 | 3 | 3 | 5 | 4 |
| 7 | 7 | 6 | 6 | 5 | 6 | 3 | 6 | 5 | 6 | 2 | 5 | 5 | 2 | 3 | 2 | 5 | 4 |
| 6 | 7 | 6 | 7 | 7 | 6 | 6 | 2 | 2 | 3 | 6 | 6 | 5 | 1 | 2 | 5 | 5 | 5 |
| 6 | 6 | 6 | 6 | 5 | 3 | 2 | 5 | 6 | 6 | 2 | 5 | 2 | 2 | 3 | 6 | 5 | 5 |
| 7 | 7 | 7 | 6 | 7 | 7 | 5 | 7 | 7 | 7 | 3 | 5 | 3 | 1 | 3 | 5 | 5 | 5 |
| 7 | 7 | 7 | 6 | 7 | 7 | 5 | 7 | 7 | 7 | 3 | 5 | 3 | 1 | 3 | 5 | 5 | 5 |
| 6 | 6 | 6 | 6 | 6 | 6 | 6 | 7 | 5 | 5 | 3 | 5 | 3 | 2 | 4 | 7 | 4 | 4 |
| 7 | 7 | 6 | 6 | 6 | 6 | 5 | 2 | 2 | 2 | 6 | 6 | 7 | 1 | 3 | 5 | 4 | 3 |
| 7 | 7 | 7 | 6 | 5 | 6 | 6 | 3 | 2 | 2 | 7 | 7 | 6 | 1 | 3 | 5 | 4 | 3 |
| 6 | 6 | 6 | 7 | 6 | 6 | 7 | 2 | 2 | 2 | 7 | 7 | 7 | 1 | 3 | 2 | 5 | 4 |
| 6 | 6 | 6 | 5 | 4 | 5 | 3 | 7 | 4 | 5 | 1 | 2 | 3 | 1 | 2 | 1 | 5 | 2 |
| 6 | 5 | 5 | 6 | 7 | 6 | 7 | 5 | 3 | 2 | 6 | 7 | 6 | 1 | 3 | 2 | 6 | 5 |
| 7 | 5 | 5 | 6 | 4 | 5 | 5 | 6 | 6 | 5 | 2 | 5 | 3 | 1 | 3 | 5 | 5 | 4 |
| 6 | 6 | 6 | 7 | 6 | 6 | 5 | 6 | 5 | 5 | 2 | 5 | 3 | 1 | 2 | 2 | 6 | 5 |
| 6 | 6 | 6 | 7 | 6 | 6 | 7 | 6 | 5 | 5 | 6 | 6 | 6 | 2 | 2 | 3 | 5 | 5 |
| 5 | 5 | 5 | 7 | 5 | 6 | 5 | 5 | 4 | 5 | 2 | 4 | 3 | 2 | 3 | 3 | 5 | 5 |
| 7 | 6 | 7 | 6 | 6 | 7 | 5 | 6 | 3 | 5 | 5 | 6 | 6 | 2 | 2 | 5 | 5 | 5 |
| 6 | 7 | 6 | 5 | 6 | 6 | 4 | 5 | 5 | 6 | 4 | 6 | 5 | 1 | 2 | 2 | 5 | 5 |
| 7 | 6 | 7 | 7 | 6 | 5 | 5 | 6 | 3 | 5 | 4 | 5 | 3 | 2 | 3 | 4 | 5 | 5 |
| 6 | 7 | 6 | 5 | 6 | 6 | 4 | 5 | 5 | 6 | 4 | 6 | 5 | 1 | 2 | 2 | 5 | 5 |
| 6 | 7 | 5 | 7 | 5 | 7 | 6 | 5 | 6 | 5 | 5 | 7 | 5 | 2 | 3 | 5 | 5 | 4 |
| 2 | 4 | 1 | 3 | 1 | 1 | 1 | 7 | 7 | 7 | 1 | 6 | 4 | 1 | 2 | 5 | 3 | 5 |

| | | | | | | | | | | | | | | | | | |
|---|---|---|---|---|---|---|---|---|---|---|---|---|---|---|---|---|---|
| 7 | 6 | 5 | 7 | 5 | 6 | 5 | 6 | 4 | 5 | 2 | 4 | 3 | 2 | 2 | 2 | 5 | 5 |
| 5 | 7 | 6 | 7 | 6 | 5 | 6 | 3 | 2 | 1 | 5 | 6 | 7 | 2 | 2 | 2 | 5 | 5 |
| 7 | 6 | 5 | 6 | 6 | 5 | 7 | 3 | 2 | 1 | 5 | 6 | 7 | 2 | 2 | 2 | 5 | 5 |
| 6 | 6 | 6 | 7 | 5 | 6 | 5 | 4 | 4 | 4 | 4 | 5 | 5 | 1 | 2 | 5 | 4 | 4 |
| 7 | 6 | 7 | 7 | 5 | 6 | 7 | 6 | 6 | 7 | 5 | 6 | 7 | 1 | 2 | 5 | 5 | 4 |
| 7 | 7 | 6 | 7 | 7 | 6 | 5 | 3 | 3 | 2 | 3 | 5 | 4 | 2 | 2 | 2 | 5 | 4 |
| 7 | 7 | 6 | 7 | 7 | 6 | 6 | 7 | 4 | 7 | 5 | 7 | 6 | 1 | 4 | 5 | 5 | 5 |
| 6 | 7 | 6 | 6 | 6 | 6 | 6 | 4 | 3 | 4 | 5 | 5 | 3 | 2 | 3 | 5 | 3 | 4 |
| 6 | 7 | 6 | 6 | 6 | 7 | 7 | 7 | 6 | 6 | 4 | 7 | 6 | 2 | 2 | 5 | 5 | 5 |
| 4 | 5 | 5 | 6 | 6 | 4 | 5 | 6 | 5 | 4 | 7 | 6 | 5 | 1 | 3 | 2 | 5 | 5 |
| 6 | 5 | 6 | 6 | 5 | 6 | 5 | 6 | 4 | 5 | 5 | 5 | 6 | 2 | 3 | 2 | 5 | 5 |
| 6 | 3 | 2 | 6 | 5 | 5 | 4 | 6 | 6 | 6 | 2 | 3 | 4 | 2 | 2 | 3 | 5 | 4 |
| 6 | 5 | 5 | 6 | 5 | 6 | 6 | 5 | 6 | 5 | 6 | 5 | 4 | 1 | 3 | 2 | 5 | 5 |
| 6 | 7 | 6 | 6 | 6 | 6 | 6 | 4 | 3 | 3 | 5 | 6 | 6 | 2 | 2 | 2 | 5 | 5 |
| 6 | 7 | 6 | 6 | 6 | 7 | 7 | 7 | 6 | 6 | 4 | 7 | 6 | 2 | 2 | 5 | 5 | 4 |
| 7 | 7 | 5 | 5 | 7 | 7 | 6 | 3 | 3 | 2 | 7 | 7 | 5 | 2 | 2 | 5 | 5 | 4 |
| 5 | 5 | 5 | 6 | 5 | 5 | 5 | 6 | 6 | 6 | 3 | 4 | 4 | 2 | 2 | 2 | 5 | 4 |
| 7 | 5 | 5 | 6 | 6 | 6 | 6 | 5 | 4 | 5 | 5 | 5 | 5 | 2 | 2 | 7 | 5 | 4 |
| 7 | 7 | 7 | 5 | 7 | 6 | 5 | 5 | 1 | 1 | 2 | 5 | 1 | 2 | 1 | 1 | 6 | 1 |
| 7 | 7 | 6 | 6 | 6 | 7 | 6 | 6 | 5 | 5 | 4 | 5 | 4 | 1 | 2 | 1 | 6 | 2 |
| 5 | 6 | 5 | 7 | 6 | 6 | 7 | 5 | 3 | 2 | 1 | 5 | 2 | 2 | 2 | 3 | 3 | 3 |
| 7 | 7 | 7 | 6 | 7 | 5 | 6 | 2 | 1 | 1 | 2 | 5 | 2 | 2 | 2 | 3 | 5 | 5 |
| 6 | 6 | 7 | 6 | 5 | 6 | 5 | 7 | 7 | 6 | 4 | 5 | 4 | 2 | 4 | 2 | 5 | 5 |
| 6 | 7 | 6 | 7 | 6 | 7 | 5 | 3 | 3 | 3 | 6 | 5 | 6 | 1 | 2 | 2 | 5 | 4 |
| 6 | 7 | 6 | 7 | 6 | 7 | 2 | 3 | 6 | 5 | 1 | 5 | 5 | 2 | 3 | 5 | 4 | 3 |
| 5 | 6 | 4 | 4 | 6 | 6 | 6 | 6 | 6 | 5 | 6 | 6 | 5 | 1 | 2 | 5 | 5 | 4 |
| 6 | 6 | 7 | 5 | 7 | 5 | 6 | 4 | 3 | 3 | 5 | 7 | 6 | 2 | 2 | 2 | 5 | 5 |
| 7 | 5 | 4 | 5 | 6 | 4 | 3 | 6 | 4 | 6 | 6 | 4 | 3 | 2 | 1 | 1 | 5 | 3 |
| 7 | 7 | 6 | 7 | 5 | 6 | 5 | 7 | 7 | 7 | 3 | 6 | 4 | 2 | 3 | 2 | 7 | 5 |
| 6 | 6 | 5 | 5 | 7 | 7 | 6 | 7 | 5 | 3 | 3 | 7 | 6 | 1 | 1 | 1 | 5 | 1 |
| 6 | 5 | 6 | 6 | 6 | 6 | 5 | 7 | 6 | 7 | 5 | 6 | 7 | 1 | 3 | 5 | 2 | 3 |
| 5 | 6 | 5 | 6 | 4 | 5 | 4 | 3 | 3 | 4 | 3 | 5 | 6 | 1 | 3 | 3 | 5 | 5 |
| 7 | 5 | 5 | 6 | 7 | 7 | 6 | 5 | 3 | 5 | 6 | 6 | 5 | 2 | 2 | 6 | 5 | 5 |
| 7 | 6 | 5 | 6 | 7 | 7 | 6 | 5 | 3 | 5 | 6 | 6 | 5 | 1 | 2 | 2 | 5 | 5 |
| 6 | 5 | 5 | 6 | 6 | 7 | 6 | 2 | 2 | 1 | 6 | 5 | 6 | 1 | 3 | 5 | 4 | 4 |
| 6 | 7 | 6 | 7 | 7 | 6 | 6 | 1 | 2 | 2 | 7 | 6 | 6 | 1 | 3 | 5 | 4 | 4 |
| 6 | 5 | 6 | 6 | 5 | 6 | 6 | 3 | 2 | 2 | 6 | 5 | 5 | 1 | 3 | 5 | 4 | 5 |
| 7 | 6 | 6 | 7 | 6 | 7 | 6 | 7 | 6 | 6 | 6 | 6 | 6 | 1 | 2 | 5 | 4 | 4 |
| 6 | 6 | 4 | 6 | 6 | 5 | 6 | 5 | 5 | 6 | 5 | 5 | 5 | 1 | 3 | 5 | 5 | 4 |
| 7 | 6 | 6 | 7 | 7 | 6 | 7 | 6 | 3 | 5 | 5 | 6 | 5 | 2 | 4 | 2 | 3 | 2 |
| 6 | 5 | 6 | 7 | 7 | 5 | 6 | 4 | 2 | 3 | 5 | 5 | 6 | 2 | 3 | 5 | 4 | 5 |
| 6 | 5 | 5 | 6 | 6 | 5 | 6 | 4 | 3 | 4 | 4 | 5 | 5 | 2 | 2 | 5 | 4 | 5 |
| 6 | 7 | 5 | 6 | 6 | 5 | 6 | 6 | 3 | 2 | 5 | 6 | 5 | 1 | 3 | 2 | 5 | 5 |
| 6 | 5 | 6 | 5 | 5 | 6 | 6 | 3 | 2 | 2 | 6 | 5 | 5 | 1 | 3 | 5 | 5 | 5 |

| | | | | | | | | | | | | | | | | | |
|---|---|---|---|---|---|---|---|---|---|---|---|---|---|---|---|---|---|
| 6 | 5 | 6 | 7 | 6 | 7 | 6 | 7 | 5 | 5 | 2 | 6 | 6 | 1 | 3 | 6 | 4 | 4 |
| 5 | 5 | 6 | 6 | 6 | 6 | 5 | 3 | 2 | 3 | 6 | 5 | 6 | 1 | 3 | 5 | 5 | 5 |
| 7 | 6 | 7 | 6 | 7 | 6 | 7 | 2 | 2 | 1 | 7 | 6 | 6 | 1 | 3 | 5 | 5 | 5 |
| 6 | 6 | 5 | 5 | 5 | 6 | 5 | 3 | 2 | 3 | 6 | 5 | 6 | 1 | 3 | 5 | 4 | 5 |
| 6 | 7 | 7 | 5 | 7 | 6 | 6 | 2 | 2 | 4 | 3 | 6 | 2 | 2 | 2 | 5 | 5 | 4 |
| 5 | 6 | 7 | 5 | 7 | 5 | 6 | 1 | 3 | 2 | 5 | 6 | 7 | 2 | 2 | 2 | 5 | 5 |
| 7 | 7 | 5 | 6 | 6 | 7 | 5 | 2 | 2 | 2 | 7 | 5 | 6 | 2 | 2 | 2 | 5 | 5 |
| 6 | 6 | 7 | 7 | 6 | 5 | 5 | 3 | 2 | 3 | 5 | 6 | 5 | 2 | 2 | 5 | 5 | 4 |
| 7 | 6 | 6 | 7 | 5 | 6 | 4 | 4 | 4 | 4 | 5 | 6 | 4 | 2 | 5 | 2 | 4 | 4 |
| 5 | 6 | 7 | 5 | 7 | 6 | 5 | 6 | 3 | 5 | 3 | 6 | 2 | 2 | 2 | 5 | 5 | 4 |
| 7 | 6 | 7 | 6 | 7 | 6 | 7 | 1 | 2 | 1 | 6 | 7 | 6 | 2 | 2 | 5 | 5 | 5 |
| 7 | 6 | 7 | 6 | 7 | 6 | 7 | 2 | 1 | 2 | 6 | 7 | 6 | 1 | 2 | 5 | 5 | 5 |
| 7 | 5 | 7 | 7 | 7 | 6 | 7 | 2 | 1 | 3 | 5 | 7 | 6 | 2 | 2 | 5 | 5 | 4 |
| 7 | 5 | 7 | 6 | 7 | 6 | 7 | 2 | 1 | 3 | 5 | 6 | 7 | 2 | 3 | 6 | 6 | 5 |
| 7 | 5 | 7 | 6 | 7 | 6 | 7 | 3 | 1 | 2 | 5 | 6 | 7 | 2 | 2 | 6 | 6 | 5 |
| 6 | 7 | 7 | 6 | 7 | 7 | 5 | 6 | 3 | 5 | 5 | 6 | 2 | 2 | 2 | 6 | 5 | 4 |
| 7 | 5 | 6 | 7 | 7 | 6 | 7 | 2 | 1 | 3 | 7 | 5 | 7 | 2 | 2 | 3 | 5 | 4 |
| 6 | 6 | 5 | 6 | 6 | 7 | 3 | 6 | 5 | 7 | 3 | 5 | 3 | 2 | 2 | 4 | 4 | 4 |
| 6 | 6 | 6 | 7 | 7 | 6 | 6 | 5 | 3 | 5 | 2 | 6 | 5 | 2 | 3 | 2 | 5 | 5 |
| 6 | 7 | 7 | 5 | 7 | 5 | 5 | 5 | 1 | 3 | 7 | 6 | 5 | 1 | 2 | 2 | 5 | 5 |
| 7 | 7 | 7 | 6 | 7 | 7 | 5 | 3 | 2 | 3 | 6 | 6 | 5 | 2 | 3 | 5 | 4 | 4 |
| 5 | 7 | 6 | 5 | 5 | 6 | 7 | 5 | 2 | 1 | 5 | 6 | 7 | 2 | 2 | 2 | 6 | 5 |
| 7 | 6 | 6 | 6 | 6 | 6 | 6 | 7 | 6 | 6 | 3 | 5 | 4 | 1 | 3 | 2 | 5 | 5 |
| 7 | 7 | 6 | 7 | 6 | 5 | 6 | 6 | 5 | 6 | 1 | 3 | 1 | 2 | 2 | 5 | 5 | 4 |
| 6 | 6 | 5 | 6 | 7 | 6 | 6 | 3 | 2 | 2 | 7 | 6 | 7 | 2 | 2 | 3 | 5 | 4 |
| 6 | 5 | 6 | 6 | 4 | 5 | 6 | 5 | 5 | 5 | 3 | 5 | 4 | 2 | 4 | 8 | 3 | 4 |
| 6 | 7 | 6 | 7 | 7 | 6 | 7 | 1 | 2 | 1 | 7 | 6 | 7 | 1 | 2 | 5 | 5 | 5 |
| 7 | 6 | 7 | 6 | 7 | 6 | 7 | 2 | 1 | 2 | 7 | 6 | 7 | 2 | 2 | 5 | 5 | 5 |
| 6 | 7 | 6 | 7 | 7 | 6 | 7 | 2 | 1 | 2 | 7 | 6 | 7 | 1 | 2 | 5 | 5 | 5 |
| 7 | 7 | 7 | 6 | 7 | 6 | 6 | 3 | 3 | 3 | 6 | 6 | 7 | 1 | 3 | 5 | 4 | 3 |
| 7 | 5 | 5 | 6 | 6 | 5 | 6 | 7 | 7 | 6 | 5 | 6 | 5 | 1 | 2 | 2 | 5 | 5 |
| 5 | 7 | 4 | 6 | 6 | 4 | 7 | 2 | 4 | 2 | 4 | 6 | 4 | 2 | 2 | 3 | 5 | 5 |
| 6 | 4 | 6 | 5 | 5 | 5 | 6 | 6 | 6 | 5 | 5 | 5 | 4 | 2 | 3 | 5 | 5 | 3 |
| 6 | 7 | 7 | 7 | 6 | 7 | 5 | 7 | 7 | 7 | 3 | 6 | 3 | 2 | 4 | 3 | 5 | 4 |
| 5 | 6 | 4 | 5 | 6 | 7 | 5 | 5 | 6 | 5 | 3 | 5 | 4 | 1 | 3 | 4 | 5 | 4 |
| 7 | 7 | 7 | 6 | 6 | 7 | 5 | 7 | 7 | 7 | 5 | 5 | 5 | 1 | 2 | 5 | 5 | 5 |
| 5 | 6 | 5 | 7 | 6 | 6 | 6 | 5 | 4 | 5 | 5 | 6 | 5 | 2 | 2 | 2 | 5 | 5 |
| 6 | 5 | 6 | 5 | 5 | 6 | 5 | 5 | 4 | 5 | 5 | 6 | 5 | 2 | 2 | 2 | 5 | 5 |
| 6 | 5 | 6 | 7 | 5 | 6 | 4 | 5 | 3 | 6 | 3 | 4 | 5 | 1 | 3 | 5 | 5 | 5 |
| 6 | 6 | 5 | 6 | 5 | 5 | 5 | 7 | 5 | 6 | 5 | 5 | 5 | 1 | 2 | 4 | 5 | 3 |
| 7 | 7 | 6 | 7 | 6 | 6 | 5 | 6 | 4 | 5 | 2 | 5 | 2 | 2 | 2 | 5 | 5 | 5 |
| 2 | 5 | 3 | 6 | 3 | 6 | 5 | 7 | 7 | 7 | 1 | 5 | 2 | 2 | 2 | 5 | 4 | 1 |
| 7 | 5 | 6 | 7 | 6 | 6 | 7 | 3 | 1 | 3 | 5 | 6 | 7 | 2 | 2 | 5 | 5 | 5 |
| 6 | 6 | 4 | 6 | 7 | 6 | 5 | 5 | 2 | 5 | 2 | 6 | 2 | 2 | 2 | 1 | 5 | 1 |

| | | | | | | | | | | | | | | | | | |
|---|---|---|---|---|---|---|---|---|---|---|---|---|---|---|---|---|---|
| 7 | 6 | 6 | 7 | 6 | 7 | 7 | 3 | 1 | 2 | 5 | 6 | 7 | 2 | 2 | 2 | 5 | 4 |
| 6 | 6 | 6 | 5 | 7 | 7 | 3 | 2 | 3 | 3 | 3 | 5 | 6 | 1 | 2 | 5 | 5 | 5 |
| 6 | 7 | 7 | 5 | 6 | 3 | 3 | 6 | 2 | 6 | 2 | 6 | 3 | 2 | 2 | 5 | 5 | 4 |
| 5 | 5 | 5 | 5 | 4 | 4 | 3 | 5 | 5 | 5 | 4 | 5 | 5 | 1 | 4 | 2 | 4 | 5 |
| 7 | 7 | 7 | 7 | 7 | 7 | 5 | 4 | 4 | 3 | 5 | 6 | 6 | 2 | 2 | 5 | 5 | 3 |
| 7 | 6 | 6 | 7 | 6 | 7 | 6 | 7 | 6 | 5 | 6 | 7 | 7 | 2 | 2 | 5 | 5 | 5 |
| 6 | 7 | 6 | 5 | 7 | 6 | 7 | 3 | 2 | 3 | 5 | 6 | 5 | 1 | 2 | 2 | 5 | 5 |
| 6 | 7 | 6 | 5 | 7 | 6 | 7 | 3 | 2 | 3 | 5 | 6 | 5 | 1 | 2 | 3 | 5 | 5 |
| 7 | 6 | 5 | 5 | 7 | 6 | 7 | 3 | 2 | 3 | 5 | 6 | 5 | 1 | 2 | 5 | 5 | 5 |
| 7 | 6 | 5 | 5 | 7 | 6 | 7 | 3 | 2 | 3 | 5 | 6 | 5 | 1 | 2 | 3 | 5 | 5 |
| 7 | 7 | 5 | 6 | 7 | 7 | 5 | 5 | 4 | 5 | 4 | 6 | 5 | 2 | 3 | 2 | 5 | 3 |
| 5 | 7 | 6 | 7 | 7 | 6 | 7 | 4 | 1 | 2 | 5 | 7 | 6 | 1 | 2 | 3 | 5 | 5 |
| 7 | 6 | 7 | 6 | 6 | 7 | 6 | 2 | 1 | 3 | 1 | 6 | 3 | 1 | 2 | 5 | 5 | 4 |
| 6 | 6 | 6 | 7 | 6 | 6 | 5 | 6 | 7 | 6 | 5 | 5 | 5 | 1 | 3 | 2 | 5 | 4 |
| 5 | 6 | 6 | 3 | 5 | 6 | 4 | 5 | 5 | 4 | 4 | 5 | 5 | 2 | 1 | 1 | 3 | 3 |
| 6 | 4 | 5 | 7 | 5 | 4 | 5 | 7 | 6 | 7 | 2 | 1 | 4 | 1 | 2 | 3 | 5 | 5 |
| 7 | 7 | 6 | 7 | 6 | 6 | 5 | 7 | 5 | 5 | 1 | 5 | 2 | 1 | 2 | 2 | 6 | 5 |
| 7 | 7 | 7 | 7 | 7 | 6 | 6 | 5 | 3 | 3 | 3 | 6 | 4 | 1 | 3 | 2 | 5 | 5 |
| 5 | 6 | 5 | 7 | 6 | 5 | 6 | 4 | 3 | 5 | 6 | 7 | 6 | 1 | 3 | 4 | 6 | 5 |
| 7 | 7 | 6 | 6 | 6 | 6 | 6 | 7 | 7 | 7 | 2 | 5 | 4 | 1 | 2 | 1 | 6 | 3 |
| 6 | 6 | 6 | 6 | 7 | 7 | 3 | 3 | 3 | 3 | 1 | 1 | 1 | 1 | 2 | 1 | 7 | 2 |
| 6 | 6 | 6 | 5 | 5 | 5 | 6 | 6 | 3 | 3 | 4 | 6 | 5 | 1 | 1 | 1 | 5 | 2 |
| 7 | 7 | 6 | 7 | 6 | 6 | 5 | 4 | 3 | 4 | 2 | 5 | 2 | 2 | 2 | 4 | 6 | 4 |
| 6 | 7 | 6 | 6 | 6 | 6 | 5 | 3 | 3 | 5 | 3 | 5 | 5 | 2 | 2 | 5 | 5 | 3 |
| 7 | 6 | 5 | 6 | 5 | 6 | 5 | 6 | 5 | 6 | 3 | 5 | 6 | 2 | 2 | 1 | 4 | 4 |
| 7 | 7 | 6 | 7 | 7 | 6 | 7 | 7 | 6 | 7 | 7 | 6 | 7 | 2 | 2 | 5 | 4 | 5 |
| 5 | 5 | 6 | 5 | 6 | 6 | 4 | 4 | 4 | 4 | 4 | 5 | 5 | 1 | 2 | 5 | 5 | 5 |
| 7 | 7 | 7 | 7 | 7 | 7 | 7 | 7 | 7 | 7 | 7 | 7 | 7 | 1 | 3 | 7 | 5 | 4 |
| 6 | 6 | 6 | 6 | 5 | 6 | 5 | 6 | 6 | 6 | 3 | 6 | 2 | 1 | 1 | 9 | 5 | 2 |
| 6 | 7 | 6 | 7 | 5 | 6 | 4 | 3 | 5 | 6 | 4 | 5 | 4 | 2 | 4 | 8 | 5 | 3 |
| 7 | 6 | 7 | 7 | 7 | 5 | 4 | 5 | 4 | 4 | 3 | 4 | 6 | 2 | 2 | 9 | 6 | 5 |
| 5 | 4 | 5 | 2 | 3 | 3 | 4 | 5 | 6 | 5 | 5 | 6 | 3 | 2 | 2 | 4 | 5 | 4 |
| 6 | 5 | 5 | 6 | 5 | 5 | 5 | 4 | 4 | 4 | 2 | 3 | 2 | 1 | 3 | 3 | 5 | 5 |
| 5 | 4 | 5 | 5 | 4 | 4 | 3 | 4 | 5 | 4 | 5 | 5 | 5 | 2 | 3 | 3 | 5 | 1 |
| 6 | 7 | 7 | 7 | 6 | 7 | 6 | 5 | 5 | 6 | 4 | 6 | 4 | 2 | 4 | 2 | 3 | 4 |
| 6 | 6 | 6 | 6 | 6 | 6 | 6 | 6 | 6 | 6 | 1 | 4 | 1 | 1 | 2 | 5 | 3 | 4 |
| 6 | 7 | 6 | 6 | 5 | 6 | 5 | 4 | 4 | 4 | 2 | 4 | 2 | 2 | 2 | 5 | 4 | 4 |
| 6 | 6 | 6 | 6 | 3 | 5 | 5 | 6 | 6 | 5 | 5 | 5 | 3 | 2 | 3 | 2 | 5 | 3 |
| 6 | 7 | 6 | 7 | 7 | 5 | 4 | 6 | 5 | 4 | 3 | 5 | 4 | 2 | 1 | 1 | 5 | 1 |
| 7 | 6 | 7 | 7 | 7 | 7 | 6 | 5 | 1 | 1 | 5 | 6 | 5 | 2 | 2 | 3 | 5 | 4 |
| 6 | 5 | 5 | 3 | 4 | 5 | 3 | 5 | 5 | 5 | 5 | 4 | 6 | 1 | 4 | 5 | 3 | 3 |
| 5 | 5 | 5 | 5 | 4 | 4 | 4 | 4 | 3 | 4 | 3 | 5 | 4 | 2 | 1 | 1 | 5 | 1 |
| 5 | 5 | 6 | 4 | 4 | 5 | 6 | 6 | 4 | 5 | 2 | 4 | 2 | 1 | 2 | 1 | 5 | 1 |
| 6 | 6 | 6 | 6 | 6 | 7 | 7 | 2 | 1 | 2 | 6 | 7 | 7 | 1 | 2 | 2 | 5 | 5 |

| | | | | | | | | | | | | | | | | | |
|---|---|---|---|---|---|---|---|---|---|---|---|---|---|---|---|---|---|
| 6 | 7 | 5 | 6 | 6 | 7 | 6 | 3 | 2 | 2 | 5 | 6 | 5 | 2 | 3 | 5 | 5 | 4 |
| 6 | 7 | 7 | 7 | 6 | 7 | 6 | 2 | 1 | 2 | 7 | 7 | 6 | 1 | 2 | 2 | 5 | 5 |
| 5 | 7 | 7 | 7 | 7 | 6 | 6 | 2 | 1 | 3 | 6 | 7 | 7 | 1 | 3 | 2 | 5 | 5 |
| 7 | 6 | 7 | 7 | 7 | 7 | 7 | 3 | 1 | 2 | 6 | 7 | 7 | 1 | 3 | 2 | 5 | 5 |
| 6 | 7 | 7 | 6 | 6 | 7 | 7 | 2 | 1 | 2 | 6 | 7 | 7 | 1 | 3 | 2 | 5 | 5 |
| 6 | 7 | 7 | 6 | 4 | 6 | 6 | 7 | 6 | 6 | 2 | 3 | 2 | 1 | 2 | 6 | 5 | 5 |
| 7 | 7 | 6 | 7 | 6 | 7 | 7 | 7 | 5 | 6 | 2 | 6 | 7 | 2 | 4 | 7 | 3 | 2 |
| 4 | 5 | 5 | 6 | 5 | 5 | 4 | 4 | 3 | 4 | 4 | 5 | 5 | 2 | 1 | 1 | 5 | 2 |
| 5 | 6 | 7 | 5 | 7 | 6 | 5 | 7 | 5 | 4 | 5 | 7 | 6 | 1 | 1 | 1 | 5 | 4 |
| 6 | 7 | 7 | 6 | 7 | 6 | 7 | 2 | 1 | 2 | 5 | 7 | 7 | 1 | 3 | 2 | 5 | 5 |
| 6 | 6 | 6 | 6 | 4 | 3 | 3 | 6 | 5 | 6 | 2 | 2 | 3 | 1 | 2 | 5 | 4 | 4 |
| 7 | 7 | 6 | 6 | 7 | 6 | 7 | 2 | 1 | 2 | 6 | 6 | 7 | 1 | 3 | 2 | 5 | 5 |
| 6 | 7 | 6 | 5 | 6 | 7 | 5 | 5 | 3 | 5 | 2 | 6 | 5 | 2 | 3 | 2 | 5 | 4 |
| 5 | 6 | 6 | 6 | 5 | 5 | 5 | 6 | 5 | 5 | 3 | 6 | 6 | 2 | 1 | 1 | 5 | 2 |
| 7 | 6 | 7 | 6 | 6 | 6 | 7 | 6 | 2 | 5 | 7 | 6 | 7 | 1 | 2 | 2 | 5 | 3 |
| 7 | 7 | 6 | 7 | 7 | 6 | 7 | 3 | 2 | 2 | 7 | 6 | 7 | 1 | 3 | 2 | 5 | 5 |
| 5 | 7 | 6 | 7 | 7 | 6 | 7 | 3 | 2 | 2 | 6 | 6 | 7 | 1 | 3 | 2 | 5 | 5 |
| 5 | 4 | 4 | 5 | 4 | 5 | 3 | 5 | 5 | 5 | 3 | 5 | 4 | 2 | 2 | 1 | 2 | 1 |
| 5 | 5 | 3 | 5 | 6 | 6 | 5 | 5 | 2 | 4 | 2 | 5 | 2 | 2 | 2 | 2 | 5 | 4 |
| 3 | 5 | 4 | 3 | 5 | 5 | 4 | 5 | 4 | 4 | 2 | 5 | 5 | 2 | 1 | 1 | 5 | 1 |
| 7 | 7 | 7 | 7 | 7 | 7 | 7 | 7 | 1 | 1 | 7 | 7 | 7 | 2 | 4 | 2 | 2 | 3 |
| 6 | 7 | 6 | 6 | 5 | 5 | 3 | 5 | 4 | 5 | 3 | 4 | 4 | 2 | 2 | 6 | 5 | 5 |
| 7 | 6 | 7 | 7 | 6 | 7 | 7 | 5 | 3 | 3 | 5 | 6 | 7 | 1 | 2 | 1 | 5 | 1 |
| 5 | 6 | 6 | 7 | 6 | 7 | 6 | 3 | 2 | 3 | 7 | 6 | 7 | 1 | 3 | 2 | 5 | 5 |
| 6 | 7 | 7 | 6 | 6 | 7 | 7 | 2 | 3 | 2 | 7 | 6 | 7 | 1 | 3 | 2 | 5 | 5 |
| 6 | 6 | 7 | 7 | 6 | 7 | 6 | 3 | 1 | 2 | 6 | 7 | 7 | 1 | 3 | 2 | 5 | 5 |
| 7 | 6 | 6 | 7 | 7 | 6 | 6 | 5 | 6 | 7 | 5 | 5 | 6 | 1 | 2 | 1 | 5 | 2 |
| 6 | 7 | 6 | 2 | 6 | 5 | 5 | 7 | 6 | 7 | 2 | 7 | 7 | 2 | 2 | 5 | 5 | 4 |
| 7 | 6 | 6 | 7 | 6 | 7 | 6 | 7 | 6 | 7 | 6 | 7 | 6 | 2 | 2 | 5 | 5 | 5 |
| 7 | 6 | 6 | 7 | 6 | 7 | 6 | 7 | 6 | 7 | 6 | 7 | 6 | 2 | 2 | 5 | 5 | 5 |
| 6 | 5 | 5 | 6 | 6 | 6 | 5 | 6 | 5 | 5 | 3 | 5 | 5 | 2 | 2 | 5 | 4 | 3 |
| 7 | 6 | 6 | 7 | 6 | 7 | 6 | 5 | 5 | 5 | 7 | 6 | 5 | 1 | 5 | 3 | 5 | 5 |
| 7 | 6 | 6 | 6 | 5 | 6 | 3 | 5 | 3 | 5 | 2 | 5 | 3 | 2 | 1 | 1 | 5 | 1 |
| 5 | 4 | 5 | 5 | 4 | 5 | 4 | 6 | 6 | 6 | 3 | 6 | 4 | 2 | 2 | 4 | 5 | 4 |
| 6 | 7 | 6 | 6 | 6 | 6 | 6 | 7 | 2 | 5 | 2 | 6 | 5 | 2 | 1 | 1 | 5 | 1 |
| 6 | 5 | 5 | 7 | 3 | 5 | 3 | 5 | 6 | 6 | 1 | 5 | 1 | 2 | 3 | 9 | 3 | 4 |
| 7 | 6 | 4 | 5 | 5 | 3 | 2 | 5 | 5 | 3 | 1 | 5 | 2 | 1 | 2 | 5 | 2 | 5 |
| 6 | 6 | 7 | 6 | 4 | 5 | 3 | 6 | 6 | 7 | 2 | 5 | 3 | 1 | 3 | 5 | 5 | 5 |
| 7 | 7 | 6 | 7 | 7 | 7 | 7 | 5 | 4 | 4 | 1 | 5 | 5 | 2 | 2 | 1 | 4 | 1 |
| 6 | 6 | 6 | 6 | 6 | 6 | 6 | 5 | 5 | 5 | 4 | 2 | 2 | 2 | 3 | 6 | 5 | 4 |
| 5 | 6 | 6 | 6 | 5 | 6 | 3 | 5 | 4 | 5 | 2 | 5 | 3 | 2 | 2 | 2 | 5 | 5 |
| 3 | 4 | 3 | 2 | 2 | 2 | 2 | 3 | 4 | 4 | 3 | 4 | 4 | 1 | 1 | 1 | 4 | 4 |
| 3 | 5 | 3 | 6 | 6 | 6 | 6 | 7 | 6 | 6 | 2 | 5 | 5 | 1 | 1 | 6 | 4 | 3 |
| 6 | 5 | 5 | 6 | 6 | 5 | 5 | 6 | 4 | 4 | 3 | 5 | 5 | 1 | 1 | 1 | 5 | 2 |

| | | | | | | | | | | | | | | | | | |
|---|---|---|---|---|---|---|---|---|---|---|---|---|---|---|---|---|---|
| 6 | 6 | 6 | 4 | 5 | 5 | 4 | 6 | 6 | 6 | 3 | 6 | 2 | 1 | 2 | 1 | 5 | 1 |
| 5 | 6 | 6 | 6 | 6 | 7 | 7 | 6 | 6 | 7 | 6 | 7 | 5 | 1 | 2 | 5 | 5 | 4 |
| 7 | 6 | 5 | 6 | 5 | 6 | 3 | 5 | 3 | 6 | 3 | 5 | 5 | 2 | 2 | 2 | 5 | 3 |
| 6 | 5 | 5 | 6 | 6 | 5 | 6 | 3 | 2 | 4 | 3 | 6 | 5 | 2 | 2 | 2 | 5 | 4 |
| 4 | 6 | 5 | 6 | 5 | 6 | 5 | 3 | 4 | 5 | 2 | 6 | 3 | 2 | 1 | 1 | 5 | 2 |
| 6 | 6 | 7 | 6 | 7 | 6 | 7 | 6 | 7 | 5 | 5 | 6 | 7 | 1 | 3 | 5 | 5 | 4 |
| 7 | 7 | 7 | 7 | 7 | 7 | 7 | 6 | 4 | 7 | 1 | 7 | 7 | 1 | 2 | 2 | 5 | 5 |
| 7 | 6 | 7 | 7 | 5 | 6 | 6 | 7 | 7 | 6 | 3 | 6 | 6 | 1 | 2 | 3 | 5 | 2 |
| 4 | 5 | 4 | 2 | 1 | 1 | 1 | 4 | 7 | 7 | 2 | 2 | 2 | 2 | 1 | 1 | 4 | 3 |
| 5 | 6 | 6 | 5 | 5 | 5 | 4 | 5 | 5 | 6 | 4 | 5 | 4 | 2 | 2 | 1 | 4 | 1 |
| 7 | 7 | 7 | 6 | 7 | 7 | 7 | 2 | 1 | 2 | 5 | 6 | 6 | 2 | 2 | 5 | 5 | 4 |
| 6 | 7 | 6 | 6 | 6 | 7 | 6 | 5 | 3 | 5 | 6 | 5 | 6 | 2 | 1 | 5 | 4 | 3 |
| 4 | 5 | 5 | 5 | 5 | 5 | 6 | 5 | 5 | 6 | 2 | 4 | 3 | 2 | 2 | 1 | 6 | 1 |
| 5 | 5 | 4 | 5 | 5 | 5 | 4 | 4 | 4 | 4 | 3 | 5 | 3 | 2 | 1 | 1 | 5 | 1 |
