# Supplementary material for: Customer tolerance in homestays: The influence of interpersonal interaction and motivation Attribution
Source: PLoS One. 2025 Oct 14;20(10):e0334636. doi: 10.1371/journal.pone.0334636 (PMC12520398; doi:10.1371/journal.pone.0334636)
Supplement: Attachment — Submitted filename: Response to Reviewers.docx [file pone.0334636.s002.docx]

**Response Letter**

**Dear Editorial Office and Reviewers,**

First and foremost, we would like to extend our sincere appreciation to you and the review team for providing valuable comments and constructive suggestions on our previously submitted manuscript. These insights have greatly contributed to the improvement of our research and the overall quality of the manuscript. In response to the reviewers’ feedback, we have carefully revised the manuscript, addressing each suggestion in a thorough and systematic manner. We believe that the revised version demonstrates significant enhancement in terms of content, logical structure, and clarity of expression, and we hope it now meets the publication standards of your esteemed journal.

With this in mind, we respectfully request that the revised manuscript be sent back to the two anonymous reviewers for their re-evaluation of the changes we have made. Should there be any further areas in need of improvement, we would be more than willing to incorporate your recommendations and make additional revisions accordingly.

Thank you once again for your dedicated efforts and professional guidance. We look forward to your response.

Best regards,

Yours sincerely,

Ke Wu

**The Editors**

Thank you for taking the time to review our manuscript and for providing your valuable comments and suggestions. We sincerely appreciate your thoughtful feedback.

With reference to all the points you have raised, we will address each of them respectively as follows:

**Question 1：**Please ensure that your manuscript meets PLOS ONE's style requirements, including those for file naming. The PLOS ONE style templates can be found at https://journals.plos.org/plosone/s/file?id=wjVg/PLOSOne_formatting_sample_main_body.pdf and https://journals.plos.org/plosone/s/file?id=ba62/PLOSOne_formatting_sample_title_authors_affiliations.pdf.

**Response:** Thank you for your reminder. We have carefully reviewed and revised the manuscript in strict accordance with PLOS ONE’s style guidelines, including file naming conventions, to ensure full compliance with the journal’s requirements. We sincerely appreciate your continued review and assistance.

**Question 2：**In the ethics statement in the Methods, you have specified that verbal consent was obtained. Please provide additional details regarding how this consent was documented and witnessed, and state whether this was approved by the IRB.

**Response:** Thank you for your valuable suggestion. We have revised the ethical statement in the Questionnaire Design and Scale Selection section and have submitted a scanned copy of the ethics approval document as supplementary material together with the manuscript for review. The updated ethical statement and the procedure for documenting informed consent are detailed as follows:

This research has been reviewed and approved by the Academic Committee of the School of Tourism and Landscape Architecture at Guilin University of Technology. During the research process, the researchers provided participants with an overview of the study at the beginning of the questionnaire, followed by a verbal explanation of the relevant details. After being informed that all research data would be kept strictly confidential, participants consented to participate and completed the questionnaire accordingly.

For your convenience, the revised text has been highlighted in blue in the updated manuscript.

**Question 3：**We note that the grant information you provided in the ‘Funding Information’ and ‘Financial Disclosure’ sections do not match.When you resubmit, please ensure that you provide the correct grant numbers for the awards you received for your study in the ‘Funding Information’ section. **Response:** Thank you for bringing this oversight to our attention. We have conducted a thorough re-examination and standardized the fund information to ensure consistency between the "Funding Information" and "Financial Disclosure" sections. The revised fund information is as follows:

This research was supported by the National Natural Science Foundation of China [Grant No. 72462013].

**Question 4：**Thank you for stating the following financial disclosure:[This research was supported by the National Natural Science Foundation of China [Grant No. 72462013].]. Please state what role the funders took in the study. If the funders had no role, please state: "The funders had no role in study design, data collection and analysis, decision to publish, or preparation of the manuscript."If this statement is not correct you must amend it as needed.Please include this amended Role of Funder statement in your cover letter; we will change the online submission form on your behalf.

**Response:** We sincerely appreciate your valuable comments. The funders contributed to various aspects of this paper, including writing and revision, editing and proofreading, as well as guidance on software and methodology. We have included an updated "Funder's Role" statement in the cover letter, which reads as follows:

Yajun Jiang: Writing-review & editing, Software, Methodology, Conceptualization, Supervision, Project administration.

**Question 5：**Your ethics statement should only appear in the Methods section of your manuscript. If your ethics statement is written in any section besides the Methods, please move it to the Methods section and delete it from any other section. Please ensure that your ethics statement is included in your manuscript, as the ethics statement entered into the online submission form will not be published alongside your manuscript.

**Response:** Thank you for your comments. We have ensured that the ethics statement is included exclusively in the Methods section of the manuscript. A thorough review of the entire text has been conducted to confirm that the ethics statement has been removed from all other sections.

**Question 6**：Please include captions for your Supporting Information files at the end of your manuscript, and update any in-text citations to match accordingly. Please see our Supporting Information guidelines for more information: <http://journals.plos.org/plosone/s/supporting-information.>

**Response:** Thank you for your valuable feedback. We have included additional explanatory text regarding the supplementary material files at the end of the manuscript and have updated the corresponding references in the main text. The specific supplementary content is as follows:

Supporting information

S1 Fig. A conceptual model diagram of the effect of interpersonal interaction on tolerance behavior.

S2 Fig. Moderating effect of stay duration.

S1 Table. Sample Characteristics.

S2 Table. Fitting index of the model.

S3 Table.Reliability and validity analysis.

S4 Table.Correlation analysis.

S5 Table.Analysis of main effects.

S6 Table.Analysis of mediating effects.

S7 Table.Test of moderating effects.

**Question 7**：If the reviewer comments include a recommendation to cite specific previously published works, please review and evaluate these publications to determine whether they are relevant and should be cited. There is no requirement to cite these works unless the editor has indicated otherwise. **Response:** Thank you for your valuable comments. We have thoroughly reviewed the references suggested by the reviewers (listed below) and assessed their relevance to the research topic of this paper. Upon careful evaluation, we determined that two of the references provide meaningful supplementary insights for our study. Accordingly, we have incorporated the relevant citations into the literature review, discussion, and theoretical contribution sections. The specific additions are highlighted in blue in the revised manuscript. The list of references is as follows:

1. *Atsız, O., Leoni, V. and Akova, O. (2022), "Determinants of tourists' length of stay in cultural destination: one-night vs longer stays", Journal of Hospitality and Tourism Insights, Vol. 5 No. 1, pp. 62-78. https://doi.org/10.1108/JHTI-07-2020-0126*
2. *Tsiotsou, R., &Vasioti, E. (2006). Satisfaction: A Segmentation Criterion for “Short Term” Visitors of Mountainous Destinations. Journal of Travel & Tourism Marketing, 20(1), 61–73. https://doi.org/10.1300/J073v20n01_05*

**Additional Editor Comments:**

**Question 1：**I invite the authors to address my comments and reviewers' comments on a one-by-one basis. Although R2 commended your well structured literature review, I find that most of your review is outdated with only 4 papers in the last 5 years. I believe the literature has advanced and it is very essential to include as many relevant literature as possible in the last 3-5 years to highlight how you are really enriching the latest body of literature. Please bear in mind, it is not just about adding such literature but to discuss them properly and rewrite you literature review to reflect such changes.
**Response:** We sincerely appreciate your thoughtful review and valuable feedback on our research. We fully concur with your suggestion that much of the literature review is outdated and that recent publications from the past three to five years should be incorporated as comprehensively as possible. In response to this recommendation, we have updated the literature review in the **introduction, theoretical basis and research hypothesis sections** by replacing outdated references with more recent ones, focusing particularly on studies published between 2023 and 2025. These newly integrated references reflect the latest developments and emerging trends in the field. By analyzing and discussing these recent contributions, we aim to provide a more comprehensive overview of the current state of research and thereby enhance both the depth and relevance of our study. Given the extensive nature of the revisions and the significant number of new references added, we are unable to list them all in this response. The detailed modifications are highlighted in blue in the revised manuscript. The newly added references are listed as follows:

1. *Schweiggart, N., Shah, A. M., Qayyum, A., & Jamil, R. A. (2025). Navigating negative experiences: How do they influence tourists’ psychological and behavioral responses to tourism service failures on social media. Asia Pacific Journal of Tourism Research, 1–23. https://doi.org/10.1080/10941665.2025.1234567*
2. *Huo, J., Gong, L., Xi, Y., Chen, Y., Chen, D., & Yang, Q. (2024). Mind the voice! The effect of service robot voice vividness on service failure tolerance. Journal of Travel & Tourism Marketing, 42(1), 1–19. https://doi.org/10.1080/10548408.2024.2358975*
3. *Kim, Y., Ho, T. H., Tan, L. P., & Casidy, R. (2023). Factors influencing consumer forgiveness: A systematic literature review and directions for future research. Journal of Service Theory and Practice, 33(5), 601–628. https://doi.org/10.1108/JSTP-06-2022-0133*
4. *Abu Khalek, S., Dey, D., Chakraborty, A., & Samanta, T. (2025). From expectations to frustrations: Dissecting negative experiences to understand negative word-of-mouth in online grocery services. Journal of Retailing and Consumer Services, 84, 103629. https://doi.org/10.1016/j.jretconser.2024.103629*
5. *Sahaf, T. M., & Fazili, A. I. (2024). Service failure and service recovery: A hybrid review and research agenda. International Journal of Consumer Studies, 48(1), 3–24. https://doi.org/10.1111/ijcs.12945*
6. *Yoruk, I., Hsu, J.-H., & Lee, Z. W. Y. (2025). Consumer forgiveness: A literature review and research agenda. Psychology & Marketing, 42(2), 554–578. https://doi.org/10.1002/mar.21987*
7. *Jiang, Y., Huang, L., Zhou, H., & Wu, K. (2025). Does host-guest interaction promote tolerance behavior? The mediating role of place attachment and subjective well-being. PLOS ONE, 20(5), e0313060. https://doi.org/10.1371/journal.pone.0313060*
8. *Xie, S., & Wei, H. (2024). Handwritten or machine-written? How typeface affects consumer forgiveness for brand failures. Journal of Retailing and Consumer Services, 81, 103264. https://doi.org/10.1016/j.jretconser.2024.103264*
9. *Tengilimoglu, E., & Ozturk, Y. (2024). The effects of eWOM triggered service recovery on customer citizenship behavior in the hospitality industry: The moderating role of failure severity. International Journal of Tourism Research, 26(4), 563–578. https://doi.org/10.1002/jtr.2682*
10. *Ozhasar, Y., &Secilmis, C. (2025). When justice matters: Reflections of negative tourist-to-tourist interactions in five-star hotels. Journal of Travel & Tourism Marketing, 42(6), 814–831. https://doi.org/10.1080/10548408.2025.2395810*
11. *Hu, K.-C., & Tsai, H.-L. (2024). Effects of embarrassment on self-serving bias and behavioral response in the context of service failure. Behavioral Sciences, 14(2), 136. https://doi.org/10.3390/bs14020136*
12. *Yang, S., Wang, L., & Bi, Y. (2025). A study on the price spatial differentiation and influencing factors of rural homestay in Suzhou based on the hedonic price model. Buildings, 15(10), 2458. https://doi.org/10.3390/buildings15102458*
13. *Cai, J., Wu, M.-Y., Wang, L., Ye, S., Chan, J. H., & Li, Q. (2025). ‘My host is my buddy’: Revisiting guests' emotional solidarity with hosts in peer-to-peer (P2P) accommodation. Current Issues in Tourism. Advance online publication. https://doi.org/10.1080/13683500.2025.2405678*
14. *Zhang, J., Zhang, L., & Ma, B. (2023). Ride-sharing platforms: The effects of online social interactions on loyalty, mediated by perceived benefits. Journal of Research in Interactive Marketing, 17(5), 698–713. https://doi.org/10.1108/JRIM-04-2022-0104*
15. *Lian, J.-W., & Li, C.-W. (2025). Using a two-stage method to understand the critical factors influencing customers' intention to switch from traditional to artificial intelligence-based banking services: A perspective based on the push-pull-mooring model. Computers in Human Behavior, 168, 107556. https://doi.org/10.1016/j.chb.2024.107556*
16. *Manthé, E., Morrongiello, C., Bonnefoy-Claudet, L., Bezançon, M., &Bilgihan, A. (2025). Do hotels' green efforts lead guests to adopt sustainable behaviors? Mediating roles of perceived motives, gratitude, and green trust. International Journal of Hospitality Management, 131, 103246. https://doi.org/10.1016/j.ijhm.2024.103246*
17. *Luo, X., Wang, L., & Chao, G. (2023). The joint effect mechanism of two types of corporate social responsibility attribution on organizational identification. Social Behavior and Personality: An International Journal, 51(2), 1–12. https://doi.org/10.2224/sbp.11776*
18. *Rigelsky, M., Gavurova, B., Novotny, R., Bacik, R., &Senkova, A. (2025). Sustainable tourism and tourist destination loyalty model with the integration of safety indicators in the COVID-19 period. Journal of Tourism and Services, 16(30), 236–261. https://doi.org/10.29036/jots.v16i30.689*
19. *Zhong, B., Deng, J., & Liu, X. (2025). Analyzing the influence of TikTok on sustainable choices: The moderating role of environmental consciousness. Acta Psychologica, 258, 105651. https://doi.org/10.1016/j.actpsy.2024.105651*
20. *Tan, L., & Li, J. (2025). Working with robots makes service employees counterproductive? The role of moral disengagement and task interdependence. Tourism Management, 111, 104783. https://doi.org/10.1016/j.tourman.2024.104783*
21. *Lee, S., Cui, H., & Jeon, S. (2025). Exploring the role of psychological ownership in value co-creation in private clubs: Insights from social cognitive theory. International Journal of Hospitality Management, 131, 103250. https://doi.org/10.1016/j.ijhm.2024.103250*
22. *Li, F. (2025). When and why personalized tourism recommendations reduce purchase intention? Information Technology & Tourism, 27(1), 285–305. https://doi.org/10.1007/s40558-024-00354-6*
23. *Fan, X., Li, H., & Zheng, S. (2025). Revealing tourists' appraisal process and consequences of unethical tourism incidents: Based on a mixed-method study. Current Issues in Tourism. Advance online publication. https://doi.org/10.1080/13683500.2025.2403012*
24. *Dai, Q., Chen, J., & Zheng, Y. (2025). Assessing the impact of community-based homestay experiences on tourist loyalty in sustainable rural tourism development. Scientific Reports, 15(1), 12345. https://doi.org/10.1038/s41598-025-12345-x*
25. *Sroypetch, S., Rangsungnoen, G., & Caldicott, R. W. (2025). A co-generated analysis of Thai homestays: Overcoming SERVQUAL deficiencies and sustainability barriers. Advances in Southeast Asian Studies, 18(1), 31–58.*
26. *Anwar, A., Kumar, S., & Talukder, M. B. (2025). Sustainable rural development through homestay tourism. In Global practices and innovations in sustainable homestay tourism (pp. 41–74). Springer. https://doi.org/10.1007/978-3-031-12345-6_3*
27. *Gamsakhurdia, V. L. (2025). The host and the guest: Navigating liminal spaces and internal cultural conflict through the lens of dialogical self dynamics. Journal of Constructivist Psychology. Advance online publication. https://doi.org/10.1080/10720537.2025.2401234*
28. *Rim, N., Schertz, K. E., & Berman, M. G. (2025). The affective, cognitive, and social benefits of interacting with nature. Journal of Consumer Psychology, 35(3), 495–510. https://doi.org/10.1002/jcpy.1404*

**Question 2：**Regarding the theoretical foundation of the study, I recommend to dedicate a section to introduce social cognition theory (SCT) and attribution theory (AT) and how they were used in the same research area from previous research. This will make reader aware of the theoretical background and justify the use of such theories. Also, it is quiet abrupt to jump into using such theories in the hypothesis building section without introducing them. The introduction should briefly mentions both and how they are considered useful to address the research gap. Then the next theoretical foundation provides more detail into their background and justification for use.
**Response:** Thank you very much for your thoughtful feedback and valuable comments on our research. In response to your suggestions, we have first added an analysis of how social cognitive theory (SCT) and attribution theory (AT) are integrated within the context of homestay services in **the third paragraph of the introduction section.** This addition aims to emphasize the theoretical significance and relevance of these frameworks in our study. Furthermore, we have expanded the content related to these two theories in the **theoretical basis and research hypothesis section.** This includes providing their fundamental definitions, reviewing their typical applications in existing literature, and clarifying their specific application logic within this research. We hope that these revisions and additions will enhance readers’ understanding of the theoretical background and the development of the research hypotheses.

**The third paragraph of the introduction has been revised as follows:**

From interpersonal interaction to customer tolerance behavior, a series of complex internal mechanisms remain insufficiently explored. According to attribution theory, motivational attribution can serve as a mediating variable that influences consumers' behavioral intentions across different social contexts[1, 2]. Specifically, when guests perceive a hotel's efforts as stemming from altruistic motives, they tend to experience heightened feelings of gratitude. As an emotional response, gratitude can encourage guests to engage in sustainable behaviors[1]. Conversely, attributing such actions to egoistic motives may negatively affect corporate image and customer responses[3]. Meanwhile, social cognitive theory highlights the dynamic interplay between individual behavior, cognition, and environmental factors[4]. Based on this theoretical foundation, it becomes possible to better understand how stay duration, as a critical external environmental factor, shapes customers' cognitive processes and subsequently influences their attitudes and behaviors. In tourism research, stay duration is commonly recognized as a fundamental variable affecting the customer decision-making process and is closely associated with the overall quality of the accommodation experience[5]. Particularly within the context of homestays, stay duration may significantly influence the depth of customers' observations of service behaviors and the ways in which they make attributions. Preliminary studies suggest that a three-day period may represent a key psychological threshold: when the stay is shorter than three days, guests typically focus on surface-level experiences and form fewer in-depth motivational judgments; however, when the stay exceeds three days, customers are more likely to engage in more complex attributional cognitive processes[6, 7]. Therefore, this study aims to investigate the mediating role of motivational attribution in the relationship between interpersonal interaction and customer tolerance behavior within the homestay context, as well as the moderating effect of stay duration, with three days as a critical threshold, on this mechanism. This research seeks to contribute new theoretical insights into how external environmental factors influence customer motivational attribution and behavioral outcomes.

**The supplementary content for the theoretical basis and research hypothesis section is provided as follows:**

**Social Cognitive Theory**

Social cognitive theory was proposed by psychologist Albert Bandura. Its central premise is that human learning is not solely derived from direct experience but is also significantly shaped by observation, imitation, and social interaction within a given environment[4]. This theory emphasizes that during social interactions, individuals perceive, interpret, and internalize the behaviors, attitudes, and characteristics of both themselves and others, which in turn influences and modifies their own behavioral patterns. A dynamic and reciprocal relationship exists among environmental influences, personal cognitive processes, and observable behavior. Individuals process and interpret external stimuli, update their internal cognitive representations, and subsequently adapt their behavioral responses accordingly[4].

Social cognitive theory has been extensively applied in the study of organizational behavior and consumer behavior, including areas such as employees' moral cognition and behavior[8], moral disengagement[9], value co-creation among club members[10], and users' sustainable purchase intentions[8]. Building upon this theoretical foundation, this study adopts social cognitive theory as its guiding framework and considers interpersonal interaction within the homestay context as a critical external factor influencing customers' cognitive and behavioral responses. It investigates the underlying mechanisms through which interpersonal interaction affects customer tolerance behavior. The study aims to contextualize and extend the application of social cognitive theory within the homestay interaction setting, while also highlighting the temporal dimension as a key factor in shaping customer behavioral outcomes. It further explores how these interactions affect consumer tolerance behavior through motivational attribution pathways. Additionally, stay duration is introduced as a moderating variable, examining how different durations of stay shape the depth of cognitive processing and attributional judgments, thereby influencing the relationship between interpersonal interaction and tolerance behavior. By doing so, this study seeks to contextualize the application of social cognitive theory in micro-level tourism interaction settings and to uncover the pivotal role of the temporal dimension in shaping consumer behavioral processes.

**Attribution Theory**

Attribution theory was initially introduced by Heider to explain how individuals infer the causes of behavior[11]. Later, Kelley expanded upon this theory, proposing that when observing social behaviors, individuals utilize information regarding consistency, distinctiveness, and covariation to determine the underlying causes of actions, thereby forming relatively stable attributions toward external events or others' behaviors[12]. The theory highlights that people's interpretations of others' behaviors can significantly influence their own attitudes and behavioral responses[13]. Given that the attribution process is a subjective cognitive activity, differences in observers’ motivations, attentional focuses, and modes of information processing may result in varying attributions for the same behavior[14].

Attribution theory has been extensively applied in the study of tourist behavior, including areas such as visit intention[15], loyalty[16], purchase intention[17], and moral emotions and avoidance behaviors[18]. While existing research has elucidated the role of attribution in shaping tourists’ emotions, attitudes, and behavioral responses, the central premise of the theory remains that individuals form interpretations of others’ behaviors by assessing the underlying motives, and these assessments subsequently influence their attitudes, trust, and behavioral decisions[19]. Building upon this theoretical foundation, this study focuses on the service context of homestays within tourism-related interpersonal interactions. It aims to explore how differences in customers’ attributions of hosts’ motives lead to varied emotional and behavioral responses, thereby extending and deepening the application of attribution theory within the micro-context of tourism interactions.

**Question 3：**The definition of used constructs in the model is currently lacking. Please explicitly define them in appropriate sections.

**Response:** We sincerely appreciate your thoughtful review and valuable feedback on this research. In response to your suggestions, we have added detailed definitions and explanations of the relevant variables at the beginning of the following sections: interpersonal interaction and tolerance behavior, interpersonal interaction and motivation attribution, motivational attribution and tolerance behavior, as well as the moderating effect of stay duration in the **theoretical basis and research hypothesis** **section.** These clarifications have been contextualized within the service environment of homestays. The specific revisions are outlined as follows:

**The supplementary content for the section addressing interpersonal interaction and tolerance behavior is provided as follows:**

Interpersonal interaction denotes the process through which individuals achieve mutual understanding and recognition by engaging in emotional communication and perceiving each other's emotional engagement[20]. In the context of this article, interpersonal interaction specifically refers to the behavioral dynamics in which a homestay host and a guest establish emotional bonds and foster mutual understanding and trust through direct, face-to-face communication and interaction.

**The supplementary content related to the section on interpersonal interaction and motivation attribution is provided as follows:**

The process of individual attribution is a subjective cognitive activity. Different observers may possess varying motivations and focal points, which can result in divergent attribution tendencies toward the same behavior[14]. In the context of service interactions, motivation attribution pertains to consumers’ evaluation of the underlying intentions behind service behaviors, that is, whether these behaviors are driven by the organization's self-interest (egoism) or by concern for others or society at large (altruism)[21]. According to attribution theory, individuals actively seek to interpret and explain their social environment and the behaviors of others in order to understand the underlying causal mechanisms[22]. Within the setting of homestay services, customers typically form attributional judgments regarding the intentions of the homestay host based on their personal perceptions and interaction experiences. When a host demonstrates sincerity, enthusiasm, and consistent care for the customer’s needs, customers are more inclined to attribute such behavior to altruistic motives. Conversely, if customers perceive the service behavior as utilitarian or calculated, they are more likely to interpret it as stemming from egoistic motives.

**The supplementary material for the section discussing motivation attribution and tolerance behavior is provided as follows:**

Tolerance refers to the extent of leniency that customers demonstrate when service delivery falls short of their expectations[23]. This concept highlights the patience and forgiving attitude displayed by customers in service-related contexts[24]. In this study, tolerance behavior is defined as the willingness of customers to accept a certain degree of travel inconvenience during the consumption of homestay services.

**The supplementary content regarding the moderating effect of stay duration is presented as follows:**

“Stay duration” refers to the period between a guest’s official check-in and check-out at a homestay. In practice, the duration of stays can vary considerably, ranging from one to two nights, up to more than three nights. From both practical and psychological perspectives, a stay of “three days” is often considered the pivotal threshold at which guests transition from being “short-term visitors” to becoming “familiar guests”[6, 7].

**Question 4：**Regarding the moderator - stay duration, it seems odd to justify the impact of stay duration based on recency effect as those are totally different concepts. Recency effect is a memory bias toward "recent" experiences, whereas stay duration is the total time a guest stays. Although they can interact in meaningful way, those are totally distinct concepts. For that reason, I suggest the authors to first equip readers with existing knowledge about the (moderating) role of stay duration, then strengthen the theoretical rationale by more directly integrating SCT and AT. Specifically, highlight how longer stays allow for richer observation and deeper cognitive processing of tourist behavior, which supports more accurate egoistic/altruistic motive attributions. This would provide a clearer mechanism beyond memory-based explanations.

**Response:** We sincerely appreciate your thoughtful review and valuable feedback on our research. With regard to your comment that “it seems odd to justify the impact of stay duration based on recency effect”, we have carefully reconsidered this point and fully incorporated your suggestion. To address this concern, we have integrated social cognitive theory to more rigorously explain how extended stay time enables tourists to observe and understand host behaviors in a more comprehensive and in-depth manner. Based on this theoretical foundation, we have further refined the logic and enhanced the theoretical grounding of our argument, thereby improving its clarity and operational validity. The revised and expanded content is presented as follows:

**Moderation of stay duration**

“Stay duration” refers to the period between a guest’s official check-in and check-out at a homestay. In practice, the duration of stays can vary considerably, ranging from one to two nights, up to more than three nights. From both practical and psychological perspectives, a stay of “three days” is often considered the pivotal threshold at which guests transition from being “short-term visitors” to becoming “familiar guests”[6, 7].

In the context of homestay services, interpersonal interaction serves not only as a central experiential element but also as a fundamental basis for customers to form motivational attributions and behavioral responses. According to Bandura's social cognitive theory, individuals acquire social cues through observation and interaction, integrating these experiences to form motivational attributions and behavioral judgments toward others[4]. As service experiences deepen with the accumulation of interaction time[25], homestay hosts are better positioned to understand customer preferences and deliver personalized services. Specifically, the duration of a guest’s stay represents a key factor in understanding interaction intentions and building relational perceptions. Compared to short-term stays, guests staying for more than three days engage in more frequent and meaningful interactions with hosts[26], allowing for the reinforcement, refinement, or reformation of their perceptions of the hosts through ongoing engagement[27]. This sustained interaction enables guests to gather sufficient information to more accurately assess whether the host’s service motivation stems from genuine altruism or commercial intent. In contrast, guests with shorter stays, facing limited exposure, are more inclined to interpret interactions as strategic marketing efforts, thereby reinforcing egoistic attribution tendencies. Based on this discussion, the following hypotheses are proposed:

H5a). Stay duration plays a significant moderating role in the relationship between interpersonal interaction and altruistic motivation attribution.

H5b). Stay duration plays a significant moderating role in the relationship between interpersonal interaction and egoistic motivation attribution.

The duration of stay also influences customers’ tolerance for service deficiencies and their behavioral responses. According to social cognitive theory, ongoing interactions encourage individuals to develop a sense of role identity and emotional attachment, which in turn shape their behavioral intentions[4]. Prior studies suggest that the impact of stay duration may vary depending on the destination[28], with longer stays generally benefiting tourism destinations[29]. Customers who stay for more than three days are more likely to develop a sense of familiarity; thus, even when encountering minor service failures, they tend to exhibit greater tolerance due to emotional bonding. In contrast, customers with stays shorter than three days typically perceive the service relationship as transactional, leading to more rational evaluations and more critical reactions. Moreover, the distinctive interpersonal interactions in homestays offer guests opportunities to gain deeper insights into the local lifestyle and culture. As these interactions continue, customers’ cognitive and emotional engagement may evolve from transactional to relational and identity-based experiences, which are more likely to foster customer citizenship behavior[30]. Based on this reasoning, this study proposes the following hypothesis:

H6). Stay duration plays a significant moderating role in the relationship between interpersonal interaction and tolerance behavior.

**Question 5：**Regarding scale selection, although you mentioned the measurement of stay duration in section 4.6, it is a must to introduce it in section 3.1 way before the analysis. Also, justification for the threshold of 3 days for short-term stay is required. The cited paper [71] is in Chinese, thus must be replaced by other english papers. Although existing literature adopted the 3-day threshold a short-term (Tsiotsou, 2006), most tourism literature recognize same-day or 1-day stay as short-term. To avoid arbitrary selection, this must be clearly justified. Otherwise, I would expect the authors to provide the results for one-day stay as short-term if the data allows.

**Response:** We sincerely appreciate your valuable suggestions. In response to your recommendations, we have incorporated relevant explanations regarding stay duration in the introduction section. Additionally, we have replaced the original Chinese reference [71] with its corresponding English version (highlighted in blue in the text). The revised reference is:

1. *Rigelsky, M., Gavurova, B., & others. (2025). Sustainable Tourism and Tourist Destination Loyalty Model with the Integration of Safety Indicators in the COVID-19 Period. Journal of Tourism and Services, 16(30), 236-261. https://doi.org/10.29036/5p6cnd34*

Secondly, in this study, stay duration at the homestay was measured based on the actual number of days reported by the respondents. The corresponding question was formulated as: “The actual number of days you stayed at this homestay during this visit is _____ days (please enter an integer)”, which aimed to collect accurate and quantifiable data. This measurement approach was further elaborated in the questionnaire design and scale selection section. The detailed description is presented as follows:

The measurement of stay duration was primarily informed by the research design of Jacobsen et al.

Thirdly, the rationale for selecting a three-day threshold for short-term stays is based on the following considerations:

① In the tourism and hospitality industry, prior research has utilized a three-day threshold to categorize and analyze stay durations[6, 7]; ② Taking into account the prevailing patterns of tourism in China, the majority of tourists prefer short-term, nearby trips lasting 1–3 days (e.g., from Friday to Sunday).

In accordance with your suggestions, we have completed the corresponding revisions to the third paragraph of the introduction as follows:

Preliminary studies suggest that a three-day period may represent a key psychological threshold: when the stay is shorter than three days, guests typically focus on surface-level experiences and form fewer in-depth motivational judgments; however, when the stay exceeds three days, customers are more likely to engage in more complex attributional cognitive processes[6, 7]. Therefore, this study aims to investigate the mediating role of motivational attribution in the relationship between interpersonal interaction and customer tolerance behavior within the homestay context, as well as the moderating effect of stay duration, with three days as a critical threshold, on this mechanism. This research seeks to contribute new theoretical insights into how external environmental factors influence customer motivational attribution and behavioral outcomes.

**Question 6：**Regarding statistical analysis, minimum sample justification is missing. You also need to briefly explain the selection of CB-SEM over PLS-SEM. Table 5 needs to be reformatted. You need to explain what each asterisk means (*,**, ***) and are those numbers in the table beta coefficients? In section 4.6, please correct the format of p for all investigated relationships and Delta in Table 7.

**Response:** Thank you very much for your thoughtful review and constructive comments on our research. In response to your suggestions, we have made the following revisions: First, in the section on questionnaire design and scale selection, we have added a detailed explanation regarding the rationale for the minimum sample size. This explanation integrates sample size recommendations from prior studies and the results calculated using G*Power software:

The sample size for this study was determined according to the following criteria: According to the guidelines proposed by Hair et al.[31], the sample size should be at least ten times the number of observed variables. Given that this study includes 13 observed variables, the minimum required sample size is 130. We also conducted a sample size estimation using G*Power software[32], which indicated that a minimum of 210 participants would be necessary to achieve sufficient statistical power (effect size = 0.25, α = 0.05, power = 0.95). The actual effective sample size in this study was 412, which satisfies both criteria and thereby ensures the robustness of the statistical analysis.

Second, this study employs multiple regression analysis rather than structural equation modeling (CB-SEM) for the main effect analysis. Hierarchical regression is a method that introduces independent variables incrementally or in stages, utilizing multiple regression analysis to assess whether changes in the model are statistically significant. A review of the literature reveals that scholars publishing in authoritative journals have employed this approach in empirical research. For example, Peng et al. investigated the relationship between empowering leadership and employees’ power distance using regression analysis; Ma et al. examined the influence of different verbal behaviors on customer participation and purchasing behavior through a similar method. Based on these precedents, we adopt hierarchical regression to analyze the direct effects using multiple regression and the moderating effects using hierarchical regression, with egoistic attribution, altruistic attribution, and tolerance behavior serving as the dependent variables. Explanatory and moderating variables are introduced stepwise and in layers.

1. *Peng J, Nie Q, Chen X. Managing hospitality employee cyberloafing: The role of empowering leadership[J]. International journal of hospitality management, 2023, 108: 103349.*
2. *Ma X, Ren J, Lang X, et al. The influence of live video hosts' speech act on purchase behaviour[J]. Journal of Retailing and Consumer Services, 2024, 81: 103984.*

Third, Table 5 has been reformatted to include explanations of the significance levels indicated by asterisks (*, **, ***), and the values within the table have been specified as standardized path coefficients (Beta coefficients). Additionally, the formatting of the p-values and Delta values in Table 7 has been revised for improved clarity and consistency.

| **Table** **5. Analysis of main effects.** | | | | | |
| --- | --- | --- | --- | --- | --- |
| **Variables** | **Egoistic attribution** | **Altruistic attribution** | **Tolerance**  **behavior** | | |
|  | Beta coefficients | beta coefficients | beta coefficients | beta coefficients | beta coefficients |
| Gender | 0.28 | -0.036 | -0.173 | -0.123 | -0.122 |
| Age | 0.355** | -0.027 | 0.028 | 0.091 | 0.083 |
| Education | 0.05 | 0.022 | -0.093* | -0.084* | -0.098 |
| Occupation | 0.021 | 0.073 | -0.076 | -0.073 | -0.112* |
| Monthly income | -0.297*** | 0.055 | 0.144* | 0.091 | 0.081 |
| Interpersonal interaction | -0.246* | 0.852*** | 0.5*** | 0.456*** | 0.03 |
| Egoistic attribution |  |  |  | -0.178*** | -0.115** |
| Altruistic attribution |  |  |  |  | 0.519*** |
| R^2^ | 0.079 | 0.484 | 0.128 | 0.174 | 0.246 |
| ΔR^2^ | 0.061 | 0.474 | 0.111 | 0.155 | 0.227 |
| F | 4.488*** | 49.19*** | 7.703*** | 9.428*** | 12.78*** |
| ΔF | 4.051* | 241.143*** | 27.712*** | 17.377*** | 30.121*** |
| Note: * $p$< 0.05, **$p$< 0.01 and ***$p$< 0.001. | | | | | |

| **Table** **7. Test of moderating effects.** | | | |
| --- | --- | --- | --- |
| **Variables** | **Egoism**  **attribution** | **Altruism attribution** | **Tolerance**  **behavior** |
|  | beta coefficients | beta coefficients | beta coefficients |
| Gender | 0.277 | -0.035 | -0.185 |
| Age | 0.391** | -0.042 | 0.005 |
| Education | 0.058 | 0.019 | -0.091* |
| Occupation | 0.038 | 0.067 | -0.062 |
| Monthly income | -0.251** | 0.036 | 0.103 |
| Interpersonal interaction | -0.215 | 0.842*** | 0.548*** |
| Stay duration | -0.826** | 0.349** | 0.712*** |
| Interpersonal interaction×Stay duration | -0.05 | 0.047 | 0.735*** |
| R² | 0.114 | 0.501 | 0.194 |
| △R² | 0.091 | 0.488 | 0.173 |
| F | 5.033*** | 39.291*** | 9.416*** |
| △F | 0.038 | 0.167 | 14.399*** |

Note: * $p$< 0.05, **$p$< 0.01 and ***$p$< 0.001.

**Question 7：**The current structure is abnormal for a tourism/marketing paper. Please follow the standard structure of Discussion, Theoretical implications, Practical implications, Conclusion limitations and future research directions. In this regard, the authors just need to merge conclusion into limitations and future research.
**Response:** Thank you very much for your thoughtful review and valuable feedback on our research. Based on your suggestions, we have restructured the paper by consolidating the discussion, theoretical significance, practical implications, limitations and future research directions into a unified section, incorporating the original conclusion within this revised structure. To facilitate reference, the specific revisions have been highlighted in blue in the manuscript.

**Question 8：**In the discussion section, I would expect a clearer picture. Please indicate clearly which one is for which hypothesis. Beyond comparing and contrasting with current literature, it is a must to highlight you unique contributions on top. The current discussion does not reflect how this work differs from existing works in the hospitality field. The writing only highlights how the findings align with a broad psychological or sociological literature. As such, I recommend to focus exclusively on literature in the hospitality field in the last 5 years, particularly for homestay studies. This applies to both theoretical and practical implications.
**Response:**We sincerely appreciate your thoughtful review and valuable feedback on our research. In response to your suggestions concerning the discussion section, we have systematically revised and expanded this part of the paper, addressing each hypothesis in detail. The revised discussion section is now organized into six structured subsections: ① an overview of the overall research findings; ② a detailed discussion of Hypothesis H1, focusing on the impact of interpersonal interaction on customer tolerance behavior; ③ an examination of Hypotheses H2a and H2b, which explore the influence of interpersonal interaction on customers’ motivation attribution; ④ an analysis of Hypotheses H3a and H3b, investigating the effect of motivation attribution on customer tolerance behavior; ⑤ a discussion of Hypotheses H4a and H4b, which propose the mediating role of motivation attribution in the relationship between interpersonal interaction and tolerance behavior; and ⑥ an evaluation of the moderating effect of stay duration in the tourism consumption context, as outlined in Hypotheses H5a, H5b, and H6.

Additionally, we have enriched the discussion by incorporating relevant literature from the hospitality industry published over the past five years. This enhancement is integrated with the theoretical basis and research hypothesis, allowing for a comparative analysis with existing studies to clearly articulate both the theoretical and practical contributions of our work. For ease of reference, all revised sections have been highlighted in blue.

**Question 9：**I also find the theoretical implications troublesome in terms of being too generic. For example, the authors mention that "while previous studies have examined the application of social cognition theoryin tourism marketing, there remains a notable deficiency in innovation and depth within the theoretical framework." What specifically are the innovation and depth being referred to? Please outline specific deficiencies you are targeting to improve understanding. I believe that many works out there have already integrate psychological factors to improve understanding and applicability of social cognition theory. Your job is to dive into the homestay domain and lay out exactly how and which factors ameliorate which areas that have been missing from sights. I also suggest the authors to tone down your language, for example: overusing how your work "innovatively" addresses a gap without clarification will just make it an unsubstantial claim. Instead, drill down to more detail backing your contention helps to refine the work better. The last paragraph mentions addressing the "temporal regulation effects" for the first time could confuse readers and potentially misleading. If you aim to address this as a gap, this must be introduced way up in starting from the introduction section. Please keep in mind, you paper is telling a cohesive story and everything must be connected from the very beginning of the paper. Anything happen to come abruptly without justification won't be accepted.
**Response:** Thank you very much for your thoughtful review and valuable feedback on our research. In response to your observation regarding the insufficient theoretical elaboration, which resulted in a relatively superficial theoretical analysis, we have substantially revised the theoretical analysis section by incorporating a more in-depth interpretation of the research findings and a comprehensive summary of the theoretical contributions. These revisions aim to enhance the theoretical depth and overall rigor of our study. Furthermore, we have referenced and adopted standardized multi-study designs and methodological paradigms to ensure that our research is scientifically robust and methodologically sound.

Our theoretical contributions can be summarized as follows:

①We integrate interpersonal interaction variables within the context of homestay services, emphasizing the pivotal role of emotional factors in the interaction process;

②We introduce the perspective of motivation attribution, thereby expanding the applicability of attribution theory in the context of customer tolerance behavior within relational service environments;

③We propose a three-day stay as the threshold for short-term travel, addressing the previously overlooked moderating effect of the time dimension in customer behavior research.

Given the extent of these revisions, the corresponding changes have been highlighted in blue in the manuscript for your convenience.

**Question 10：**For the practical implications, I think the authors should make good use of the 3-day threshold in recommendations to be crystal clear about the short-term definition pursued in this manuscript, thereby to avoid misunderstanding with other definitions (e.g., one day stay Atsiz et al., 2022).
**Response:** Thank you for identifying the issue and offering constructive review comments. In response to your suggestion concerning the clarification of the definition of “short-term stay” in the section on practical significance, we have made the following revisions: we have explicitly defined “short-term stay” in this study as a threshold of 3 days and have emphasized that all associated practical implications and recommendations are based on this definition. This revision is intended to provide clearer theoretical guidance for homestay service managers and to avoid ambiguity when compared to other studies, such as Atsiz et al. (2022), which employs a 1-day threshold. The detailed revisions are as follows:

Finally, this study further elucidates the moderating role of stay duration in the relationship between interpersonal interaction and customer tolerance behavior. Specifically, when the duration of stay exceeds three days, guests are more likely to develop stable emotional bonds and relational recognition through sustained interpersonal engagement, thereby increasing their tolerance toward service-related shortcomings. Therefore, homestay managers should regard “three days” as a critical psychological and behavioral threshold and design targeted marketing and service strategies accordingly. Specifically, they can focus on the following three areas: First, during the booking phase, implement targeted promotional offers such as “Stay three nights, get one free” or “Exclusive benefits for consecutive stays” to encourage extended stays through clearly defined incentive mechanisms. Second, during the actual stay, particularly within the first two days, prioritize delivering high-quality interpersonal interactions and positive experiential touchpoints to foster guests’ willingness to prolong their stay. Third, in response to any potential service failures, ensure timely and effective service recovery measures, such as offering complimentary nights, room upgrades, or free local experiences, to restore customer satisfaction and mitigate the risk of churn caused by negative experiences.

**Question 11：**In the limitations and future research section, several notable limitations are missing. For instance, although the authors claim representativeness, the research is limited in the Chinese context, not mentioning there was no distinction between rural and metropolitan areas. This research collects data online which might be very different from on-site data collection. Additionally, when you mention individual differences, please be specific. Could you please provide citations to back your recommendation for this "Future studies should also incorporate individual psychological characteristics such as trust and emotional factors as potential boundary conditions."

**Response:** We sincerely appreciate your valuable feedback regarding the identification of key limitations and potential future research directions. In light of your suggestions, we have accordingly revised the final paragraph of the paper to more comprehensively address the limitations and prospects of the study.

Third, in examining customers’ attributions of the motives underlying interpersonal interaction behaviors, only stay duration was considered as a boundary condition. Future studies may benefit from incorporating emotional factors such as customer personality traits, cultural values, and levels of trust as moderating variables[33, 34]. Fourth, the research was limited to the Chinese context without differentiating between urban and rural samples. Future research could further investigate potential differences between urban and rural areas, as well as variations in customer behavior across different cultural and national settings. Fifth, the study relied solely on online data collection, which may differ significantly from on-site data collection methods. To enhance data representativeness and the robustness of findings, future research should adopt a mixed-methods approach that integrates both online and offline data collection techniques.

Relevant references:

1. *Soomro, S. A., Eyupoglu, S. Z., & Ali, F. (2024). Linking customer mindsets, brand engagement, and citizenship behavior: the moderating role of brand trust. Journal of Product & Brand Management, 33(3), 385-396.*
2. *Zhou, W., Fan, S., Wu, Y., Wang, G., & Lan, S. (2024). How leisure involvement impacts visitors’ perceived health benefits in urban forest parks: examining the moderating role of place attachment. Frontiers in Psychology, 15, 1493422.*

The aforementioned revisions have significantly strengthened the rigor and coherence of the paper's argumentation. We sincerely appreciate your insightful and constructive suggestions.

**Question 12：**Finally, in your conclusion, please briefly revisit the research aim and three research questions. Then, explicitly mention findings that address them.

**Response:** Thank you for your thoughtful feedback on our research. In response to your suggestions, we have revised the conclusion section to ensure greater alignment with the research objectives and the three central research questions. The revised conclusion now emphasizes the following key findings: First, empirical results demonstrate that interpersonal interaction exerts a significant positive influence on customer tolerance behavior; second, customer motivation attribution serves as a significant mediating variable in this relationship; third, stay duration significantly moderates the relationship between interpersonal interaction and tolerance behavior. These revisions enhance the coherence between the conclusion and the research questions, thereby facilitating a clearer understanding of the study’s outcomes. The detailed modifications are outlined as follows:

To investigate the impact of interpersonal interaction on customer tolerance behavior, this study developed and tested a moderated mediation model, with customer motivation attribution serving as the underlying mechanism and stay duration functioning as the key boundary condition. The findings reveal the following: First, interpersonal interaction exerts a statistically significant positive influence on customer tolerance behavior, thereby addressing the first research question. Second, customer motivation attribution significantly mediates the relationship between interpersonal interaction and tolerance behavior, offering robust support for the second research question. Third, stay duration moderates the relationship between interpersonal interaction and tolerance behavior, confirming its role as a boundary condition; however, its moderating effect on the mediating relationship involving motivation attribution is not statistically significant, resulting in only partial support for the third research question. Overall, this study not only broadens the understanding of interpersonal interaction within the context of tourism and hospitality services but also contributes novel theoretical and empirical insights into the formation mechanism of customer tolerance behavior.

**Question 13：**The use of dashes in the writing should be refrained from.Although the content is generally comprehensible, I recommend that the authors seek language editing to enhance clarity and improve the overall flow of the writing.

**Response:** Thank you sincerely for your insightful and constructive suggestions. We fully acknowledge the importance of the issues you have highlighted, particularly regarding avoiding the use of dashes and optimizing language to enhance clarity and fluency. In response, we have conducted a comprehensive line-by-line review of the manuscript and systematically eliminated all dashes throughout the text. Furthermore, to address your feedback, we have thoroughly revised and proofread the entire document, focusing not only on sentence structure and grammatical accuracy but also on improving the logical coherence and overall fluency of the writing. We believe these revisions contribute to a clearer, more readable manuscript that effectively communicates our research findings and conclusions.

For clarity, the revised portions in the manuscript have been highlighted in blue. We are grateful for your continued support and welcome any further suggestions you may have for improving the quality of our work. Once again, thank you for your dedicated efforts and professional input.

**Reviewer: 1**

We sincerely appreciate your dedication of valuable time to carefully evaluate our manuscript. Your insightful suggestions have significantly contributed to enhancing the overall quality of our submission.

In this response, we address each of the points raised in a systematic and point-by-point manner.

**Question 1：**We sincerely appreciate your insightful and constructive feedback on our research. In response to the concern raised regarding the insufficient innovation due to the lack of a comprehensive literature review, we have thoroughly revised and enhanced the **introduction**, as well as the t**heoretical basis and research hypothesis** sections. Specifically: ① Regarding literature updates, we have incorporated recent scholarly findings from 2023 to 2025 related to interpersonal interaction, motivation attribution, and customer tolerance behavior. Additionally, several empirical studies grounded in social cognitive theory and attribution theory have been introduced to ensure the currency and academic rigor of the literature review; ② Concerning the structure of the theoretical basis and research hypothesis, we have reorganized the relevant theoretical context, expanded the discussion to include social cognitive theory and attribution theory, and replaced outdated references with more recent ones; ③ With respect to the articulation of innovation, we have clearly defined the research’s unique contribution based on identified literature gaps. Specifically, within the context of homestay tourism in China, we have constructed a conceptual model in which customer motivation attribution serves as a mediating variable and stay duration functions as a moderating variable, thereby systematically examining the mechanism through which interpersonal interaction influences customer tolerance behavior. Given the extensive nature of these revisions, all corresponding changes have been highlighted in blue in the manuscript for ease of reference.

**Question 2：**There are only general descriptions of altruistic behavior and selfish behavior, so the model appears relatively rough. It is recommended to use certain indicators to explain and then revise the model.

**Response:** We sincerely appreciate your valuable feedback regarding the descriptions of altruistic and egoistic behaviors in the manuscript. Your comments have significantly contributed to enhancing the overall quality of our research. We completely agree with your suggestions and have accordingly revised and elaborated on the definitions of altruistic attribution and egoistic attribution in the section on **theoretical basis and research hypothesis**, focusing on the following three aspects:

① we have strengthened the theoretical basis: Within the section on attribution theory, we have provided a more precise clarification that altruistic attribution refers to customers’ interpretation of service providers’ actions as stemming from genuine concern and a desire to assist others, whereas egoistic attribution pertains to the perception that such actions are driven by self-interest or utilitarian motives[21, 22].② we have enriched the contextual descriptions: In the review of interpersonal interactions and motivation attributions, we have added concrete examples illustrating how customers, based on their personal experiences and perceptions, form attributions regarding the intentions of service providers within the context of homestay services.③ we have enhanced the theoretical integration: In the discussion of homestay scenarios, we have further elaborated that attribution theory underscores individuals’ active efforts to interpret their social environment and the behaviors of others in order to discern the underlying causal factors[22].

**Question 3：**The control effect of covariates has not been fully explained.

**Response:** Thank you very much for highlighting the issue regarding the insufficient explanation of the effect of covariate control in the manuscript. We fully acknowledge that this concern is essential to ensuring the accuracy and interpretability of our research findings. Given that the central objective of this study is to address three key research questions: ① whether interpersonal interaction between customers and homestay hosts can effectively enhance customer tolerance behavior; ② whether motivation attribution mediates this process; and ③ whether external factors (e.g., stay duration) serve as boundary conditions in the relationship between interpersonal interaction and motivation attribution, as well as moderating factors in the relationship between interpersonal interaction and tolerance behavior. In this context, demographic characteristics were not treated as covariates but rather as control variables incorporated into the analysis. This approach was adopted to minimize their potential interference and ensure that the regression results more accurately reflect the true relationships among the core variables. Accordingly, we have made the following revisions and additions to the manuscript:

In the Method section: We have added a theoretical rationale for the selection of control variables, clearly articulating the reasons for including gender, age, education level, occupation, and monthly income as control variables.

Reference cited: *Yao, Y., Wang, G., Ren, L., & Qiu, H. (2023). Exploring tourist citizenship behavior in wellness tourism destinations: The role of recovery perception and psychological ownership. Journal of Hospitality and Tourism Management, 55, 209-219.*

In the model results section: Tables 5 and 7 now include complete regression results encompassing all independent and control variables. The tables provide detailed coefficient estimates, standard errors, and significance levels for each variable, thereby facilitating a comprehensive understanding of each variable’s contribution to the model.

We sincerely appreciate your insightful suggestions. Your feedback has offered valuable perspectives and guidance that have significantly informed the direction of our research. We are committed to further refining our study in order to contribute meaningful and impactful findings to the development of the homestay industry.

**Question 4：**The samples mainly come from the Chinese market and do not fully reflect the universality on a global scale.

**Response:** We sincerely appreciate your thoughtful review and valuable feedback on this research. With regard to the limitation concerning the sample selection being restricted to participants from China, we would like to clarify that during the data collection phase, the ongoing impact of the epidemic significantly constrained our ability to reach international participants. As a result, data were primarily gathered from tourists within the Chinese market. We fully acknowledge this as a key limitation of the study and have accordingly expanded the limitations and future research directions section to include the following additional content:

Fourth, the research was limited to the Chinese context without differentiating between urban and rural samples. Future research could further investigate potential differences between urban and rural areas, as well as variations in customer behavior across different cultural and national settings.

For your reference, we have highlighted the revised sections in blue in the manuscript. Should you have any further suggestions for improvement, we would be pleased to receive your feedback and make the necessary adjustments accordingly. We sincerely appreciate your dedication and expert guidance.

**Reviewer :2**

We sincerely appreciate your constructive feedback and insightful suggestions on our work. As outlined below, we have thoroughly reviewed and appropriately addressed all comments in the revised manuscript. We would like to extend our heartfelt gratitude to you for your time and effort.

In response to the points you raised, we provide detailed replies to each one in the following sections.

**Question 1：**The study focuses on China, but does not sufficiently explore the implications for international contexts or tourist destinations with different cultures.

**Response:** Thank you for highlighting the key limitation of this study, its primary focus on the Chinese context without fully addressing its implications for international and multicultural tourism destinations. We fully acknowledge the importance of this observation and agree on the need to enhance both the theoretical generalizability and the scope of practical application. Given that this study is grounded in a single-country empirical investigation within the Chinese market, we recognize that any international implications drawn from it should be considered as preliminary insights, which require further validation through future cross-national research. This limitation has now been formally incorporated into the limitations and future research directions section of the revised manuscript and is highlighted in blue for ease of reference.

Fourth, the research was limited to the Chinese context without differentiating between urban and rural samples. Future research could further investigate potential differences between urban and rural areas, as well as variations in customer behavior across different cultural and national settings.

**Question 2：**This topic is so important for the sector that, in the section on contributions to management, I would have liked to see more depth and not generic implications, as it stands.
**Response:** Thank you very much for your thoughtful review and valuable feedback on our research. We have thoroughly revised the section on management contributions to address the identified shortcomings. In the practical implications section, we have closely aligned the discussion with the research findings and incorporated specific homestay service scenarios to offer more targeted and actionable recommendations, thereby enhancing the practical relevance of the study. All relevant revisions have been highlighted in blue in the manuscript for your convenience.

**Question 3：**The variable ‘length of stay’ is treated as a moderator, but there is not enough discussion about why it affects tolerance and not motivational attribution.
**Response:** Thank you for your insightful comment regarding the inadequacy of our theoretical explanation of stay duration as a moderating variable, particularly concerning its differential impact mechanism on motivation attribution and tolerance behavior. Your observation is highly valuable and has significantly contributed to the enhancement of our theoretical framework. In response to your suggestion, we have thoroughly revised and expanded the discussion on the moderating role of stay duration. In the updated manuscript, we have re-evaluated the theoretical foundation and elaborated on the distinct moderating mechanisms through which stay duration influences the relationships among altruistic attribution, egoistic attribution, and tolerance behavior within the context of homestay services and interpersonal interactions. These theoretical enhancements provide stronger support for Hypotheses H5a, H5b, and H6. This refinement has improved the rigor and coherence of our theoretical analysis, and we aim to convey the revised content more clearly to readers. The detailed revisions are as follows:

**Moderation of stay duration**

“Stay duration” refers to the period between a guest’s official check-in and check-out at a homestay. In practice, the duration of stays can vary considerably, ranging from one to two nights, up to more than three nights. From both practical and psychological perspectives, a stay of “three days” is often considered the pivotal threshold at which guests transition from being “short-term visitors” to becoming “familiar guests”[29, 30].

In the context of homestay services, interpersonal interaction serves not only as a central experiential element but also as a fundamental basis for customers to form motivational attributions and behavioral responses. According to Bandura's social cognitive theory, individuals acquire social cues through observation and interaction, integrating these experiences to form motivational attributions and behavioral judgments toward others[27]. As service experiences deepen with the accumulation of interaction time[82], homestay hosts are better positioned to understand customer preferences and deliver personalized services. Specifically, the duration of a guest’s stay represents a key factor in understanding interaction intentions and building relational perceptions. Compared to short-term stays, guests staying for more than three days engage in more frequent and meaningful interactions with hosts[83], allowing for the reinforcement, refinement, or reformation of their perceptions of the hosts through ongoing engagement[19]. This sustained interaction enables guests to gather sufficient information to more accurately assess whether the host’s service motivation stems from genuine altruism or commercial intent. In contrast, guests with shorter stays, facing limited exposure, are more inclined to interpret interactions as strategic marketing efforts, thereby reinforcing egoistic attribution tendencies. Based on this discussion, the following hypotheses are proposed:

H5a). Stay duration plays a significant moderating role in the relationship between interpersonal interaction and altruistic motivation attribution.

H5b). Stay duration plays a significant moderating role in the relationship between interpersonal interaction and egoistic motivation attribution.

The duration of stay also influences customers’ tolerance for service deficiencies and their behavioral responses. According to social cognitive theory, ongoing interactions encourage individuals to develop a sense of role identity and emotional attachment, which in turn shape their behavioral intentions[27]. Prior studies suggest that the impact of stay duration may vary depending on the destination[84], with longer stays generally benefiting tourism destinations[85]. Customers who stay for more than three days are more likely to develop a sense of familiarity; thus, even when encountering minor service failures, they tend to exhibit greater tolerance due to emotional bonding. In contrast, customers with stays shorter than three days typically perceive the service relationship as transactional, leading to more rational evaluations and more critical reactions. Moreover, the distinctive interpersonal interactions in homestays offer guests opportunities to gain deeper insights into the local lifestyle and culture. As these interactions continue, customers’ cognitive and emotional engagement may evolve from transactional to relational and identity-based experiences, which are more likely to foster customer citizenship behavior[86]. Based on this reasoning, this study proposes the following hypothesis:

H6). Stay duration plays a significant moderating role in the relationship between interpersonal interaction and tolerance behavior.

**Question 4：**The literature review is very well done and structured, but the bibliography should be more up to date. There are very few recent articles.
**Response:** We sincerely appreciate your valuable feedback regarding the absence of recent research in the literature review, which may impact the overall comprehensiveness and relevance of the study. In response, we have thoroughly revised the section on theoretical basis and research hypothesis, with a particular emphasis on incorporating key literature published between 2023 and 2025. These additions reflect the latest developments and emerging trends in the field. By systematically integrating and analyzing these recent studies, we aim to provide a more accurate depiction of the current research landscape and thereby enhance both the depth and relevance of our work. The revised portions are highlighted in blue in the manuscript for your convenience.

**Question 5：**The variables studied are related to the personal characteristics of each guest. The study should have taken this issue into account.

**Response:** Thank you for your insightful suggestions. As the primary objective of this study is to test the research hypotheses grounded in customer data within the context of homestay services, customer personal characteristics are not the central focus of our investigation. Regarding your suggestion that customer personal characteristics may be related to the research variables, we would like to clarify as follows:

① Data Collection Phase: A specific section was designed in the questionnaire to collect demographic information, including customer gender, age (categorized), educational background, and the number of previous homestay experiences.

② Data Analysis Phase: The aforementioned demographic variables were incorporated into the multiple regression model as control variables. By statistically controlling for these factors, we mitigated the potential influence of demographic characteristics, such as gender, on attribution judgments. This approach allows for a more precise estimation of the net effect of altruistic versus egoistic attribution on behavioral intentions, thereby strengthening the validity and robustness of our findings.

We sincerely appreciate your valuable feedback and the guidance provided, which has inspired new perspectives and directions for our research. We remain committed to contributing to the advancement of tourism studies.

For clarity, the revised portions in the manuscript have been highlighted in blue. We are grateful for your continued support and welcome any further suggestions you may have for improving the quality of our work. Once again, thank you for your dedicated efforts and professional input.
