# Supplementary material for: Customer tolerance in homestays: The influence of interpersonal interaction and motivation Attribution
Source: PLoS One. 2025 Oct 14;20(10):e0334636. doi: 10.1371/journal.pone.0334636 (PMC12520398; doi:10.1371/journal.pone.0334636)
Supplement: Attachment — Submitted filename: Response_to_Reviewers_auresp_2.docx [file pone.0334636.s003.docx]

**Response Letter**

**Dear Editorial Office,**

First of all, we would like to thank you sincerely for your helpful comments and suggestions on our previous manuscript. Your feedback has been very helpful for us to improve our work.

Based on the additional editor’s feedback, we have revised the manuscript carefully and tried to respond to every suggestion. We believe these changes have made the manuscript clearer and better organized.

We would be very grateful if the revised manuscript could be sent back to the additional editor for re-evaluation. If there are still areas that need more improvement, we are willing to make further changes.

Thank you again for your time and guidance. We look forward to your reply.

Best regards,

Yours sincerely,

Ke Wu

**The Editors**

Thank you for taking the time to review our manuscript and for providing your valuable comments and suggestions. We sincerely appreciate your thoughtful feedback.

With reference to all the points you have raised, we will address each of them respectively as follows:

**Journal Requirements:**

**Question 1：**If the reviewer comments include a recommendation to cite specific previously published works, please review and evaluate these publications to determine whether they are relevant and should be cited. There is no requirement to cite these works unless the editor has indicated otherwise.

**Response:** Thank you very much for your guidance regarding the recommendation to cite specific published works. We carefully reviewed the reviewers’ comments and confirmed that they did not require the citation of particular references. For the works suggested by the editor, we examined their research topics, core arguments, and methodologies, and evaluated their theoretical relevance and potential contribution to our study (e.g., host–guest interactions in P2P accommodation). We also considered their academic influence and timeliness to ensure that any added citations provide substantive support for our arguments. Based on this assessment, we incorporated only those studies that are highly relevant to our research.

[1] *Atsız, O., Leoni, V. and Akova, O. (2022), "Determinants of tourists' length of stay in cultural destination: one-night vs longer stays", Journal of Hospitality and Tourism Insights, Vol. 5 No. 1, pp. 62-78. <https://doi.org/10.1108/JHTI-07-2020-0126>*
[2] *Tsiotsou, R., & Vasioti, E. (2006). Satisfaction: A Segmentation Criterion for “Short Term” Visitors of Mountainous Destinations. Journal of Travel & Tourism Marketing, 20(1), 61–73. <https://doi.org/10.1300/J073v20n01_05>*

We sincerely appreciate your guidance, which helped us clarify the standards for selecting citations.

**Question 2：**Please review your reference list to ensure that it is complete and correct. If you have cited papers that have been retracted, please include the rationale for doing so in the manuscript text, or remove these references and replace them with relevant current references. Any changes to the reference list should be mentioned in the rebuttal letter that accompanies your revised manuscript. If you need to cite a retracted article, indicate the article’s retracted status in the References list and also include a citation and full reference for the retraction notice.

**Response:** Thank you for your reminder regarding the accuracy of the reference list. We have carefully reviewed all references in the manuscript and confirm that none of the cited works have been retracted; all references remain valid publications. We will continue to adhere to the journal’s requirements and will make timely corrections should any issues arise in the future.

**Additional Editor Comments:**

**Question 1：**[Line 102-103, p.3], the authors mention that the study investigates "three primary research questions", however I could not find the three research questions but rather the research aims.

Response: Thank you very much for pointing out the inconsistency between our description of the research aims and the stated “three primary research questions.” We have revised the manuscript accordingly. Specifically, on page 3, lines 102–103, we have now clearly listed the three research questions immediately following the statement:

RQ1: Does interpersonal interaction between homestay hosts and guests significantly enhance customers’ tolerance behavior?

RQ2: Does motivational attribution mediate the relationship between interpersonal interaction and tolerance behavior?

RQ3: Does stay duration moderate the relationships between interpersonal interaction, motivational attribution, and tolerance behavior?

These revisions ensure that the research questions are explicitly presented and aligned with the overall research aims.

**Question 2：**Also, in the intro, the research aims are scattered across multiple paragraphs which make it hard for reader like me to follow through. For example, the aims can be found between [line 72-74], and then [line 95-100]. [line 102-111] should be sufficient to elicit the research aims. Other parts should be rewritten to avoid distracting readership.

**Response:** Thank you very much for your valuable comment regarding the presentation of the research aims in the Introduction. We agree with your observation that the aims were previously scattered across several paragraphs (e.g., lines 72–74 and 95–100), which may reduce clarity for readers. To address this, we have restructured the Introduction so that the research aims are now presented in a consolidated manner in lines 102–111. In addition, the preceding sections have been revised to follow a clearer sequence of “research background→research gap→theoretical foundation → research questions→contributions,” thereby avoiding repetition or distraction and ensuring a coherent flow.

In today’s service economy, tolerance has become a critical behavioral response that reflects how customers manage service shortcomings[1, 2]. Tolerance behavior demonstrates customers’ patience and forgiveness toward service failures and functions as a key psychological indicator of service evaluation[2, 3]. Although some customers respond to service deficiencies with complaints or negative word-of-mouth[1, 4, 5], tolerance allows them to restore psychological balance and creates opportunities to repair relationships[6]. Several notable scholars have examined tolerance in financial services, healthcare, retail, and hospitality[7-11]. Yet research on tolerance in homestays remains limited, even though this setting emphasizes personalized hospitality and interpersonal engagement more than standardized service systems[12].

Homestays typically operate on a smaller scale[13] and involve frequent, direct host-guest interactions. Unlike traditional hotels, they rely on informal exchanges, emotional connections, and opportunities to engage with local culture and traditions[14-16]. These unique characteristics suggest that tolerance in homestays depends not only on perceived service quality but also on interpersonal dynamics and cultural expectations. In East Asian contexts, especially, where relational orientation and emotional reciprocity hold strong value[17, 18], tolerance functions both as a behavioral adjustment and as a cultural expression. However, tourism and hospitality studies have yet to fully explain how host-guest interactions translate into tolerance behavior[19-22].

To fill this gap, we employed attribution theory and social cognitive theory. Attribution theory explains how customers’ perceptions of service providers’ motives shape their emotional and behavioral responses[23-25]. When guests interpret hosts’ behavior as altruistic, they feel gratitude and act more cooperatively[23]. In contrast, egoistic attributions reduce tolerance and weaken relationships[25]. Social cognitive theory highlights the interplay of personal, behavioral, and environmental factors[26]. In homestays, stay duration represents an important environmental factor: shorter stays often generate surface-level impressions, while longer stays allow deeper observation and more complex attributions[27]. Emerging evidence suggests that a three-day threshold may mark the transition from superficial judgments to more elaborate cognitive evaluations[28, 29].

Building on these insights, we systematically examine the psychological processes underlying customer tolerance in homestays. Specifically, we addressed the following research questions:

RQ1: Does interpersonal interaction between homestay hosts and guests significantly enhance customers’ tolerance behavior?

RQ2: Does motivational attribution mediate the relationship between interpersonal interaction and tolerance behavior?

RQ3: Does stay duration moderate the relationships between interpersonal interaction, motivational attribution, and tolerance behavior?

This study makes three key contributions. Theoretically, it integrates social cognitive theory and attribution theory to explain how interpersonal interactions foster tolerance, extending their application within tourism contexts. It also identifies stay duration as a boundary condition, highlighting the temporal dimension of cognition and behavior in homestays. Practically, it provides actionable insights for homestay operators, emphasizing the importance of nurturing emotional connections and managing guest expectations across varying lengths of stay. By clarifying these mechanisms, we advance both academic understanding and managerial practice in homestay services.

Thank you again for your detailed guidance. Your suggestions have significantly improved the information focus and readability of the introduction. If any adjustments are still needed in the logic or expression of the introduction in the future, we will make the necessary improvements immediately!

**Question 3：**Please remove all the closed parentheses for hypotheses such as H1)., instead just "H1." should suffice.Figure 1 on p.9 is missing.

**Response:** Thank you for your comment regarding the formatting and completeness of the manuscript. We have revised the numbering of all hypotheses from “H1).” to “H1.” as suggested, and we have also included the missing Figure 1 on page 9. These changes have been marked in the revised manuscript for your review.

**Question 4：**[Line 382-384] the ten-time rule is so outdated and proven wrong. Please remove that. Using G*Power should be enough.

**Response:** Thank you for pointing out the issue regarding the sample size justification. We have removed the outdated “ten-times rule” statement previously included in lines 382–384 and retained only the explanation based on G*Power 3.1. This revision has been highlighted in the revised manuscript for your review.

**Question 5：**[Line 402-403] Jacobsen et al. is lacking the year. Plus, I could not find them in the reference list. You can't cite what is not in the reference list. I request a thorough review of all in-text citations which must be in the reference list. I will double-check the next revision.I also can't verify this as well "Wang J. Dimension determination and empirical Test of host-Guest Interaction: A case study of China's homestay industry. Statistics and Information Forum. 2018;33(11):118-124." Documents in English must be prioritized to facilitate our wide readership.

**Response:** Thank you for your detailed check of the in-text citations and reference list. We have carefully revised the manuscript as follows.

First, for Jacobsen et al., we have added the publication year and included the full reference in the reference list. Second, we conducted a systematic cross-check between all in-text citations and the reference list to ensure that every citation is properly included and formatted according to the journal’s requirements. Third, to enhance accessibility for an international readership, we have replaced the Chinese reference (Wang, 2018) with a suitable English-language source, for example:

*Chen, H., Fu, S., & Lyu, B. (2024). Homestays in China: Mediator effects of brand perception upon host-guest interaction and Tourist behavior intention. Heliyon, 10(8).*

All these revisions have been marked in the revised manuscript for your review.

**Question 6：**[Line 404-406] this sentence creates unnecessary confusion. Since each scale was developed with rigor based on a certain context, adapting the scale to a different context could be understandable. However, your sentence feels like you cherry-pick each item. How exactly deleting items lead to better accuracy? Please rewrite with clarity to avoid confusion.

**Response:** Thank you for pointing out the ambiguity in our description of the scale adaptation. We agree that the original wording in lines 404–406 may have been misleading and could be interpreted as arbitrarily deleting items to increase accuracy. To address this, we have rewritten the section to clearly explain the adaptation process. Specifically, we now state that items were removed only after careful consideration of their contextual irrelevance or redundancy, and that the retained items were consistent with the theoretical framework and supported by prior validation studies. This revision provides a more transparent rationale for the adaptation and avoids any possible misunderstanding.

Next, we translated all scales from validated English versions into Chinese using a back-translation process. Tourism and hospitality researchers reviewed and refined the draft questionnaire. A pilot test with 90 university students and teachers who had recent homestay experience prompted minor revisions. These steps confirmed the questionnaire’s face validity. Also, all constructs used a 7-point Likert scale ranging from 1 (strongly disagree) to 7 (strongly agree). The final section collected demographic characteristics.

Through this revision, the methodological description is now clearer and avoids the potential misunderstanding of “arbitrary item deletion.” We remain open to further refinement if additional clarification is required.

**Question 7：**By “foreign mature scales” [line 406], what does it mean to be specific? In Table 1, please replace all “~” by “-”.

**Response:** Thank you for your precise suggestions regarding terminology and formatting. To avoid ambiguity, we have revised the phrase “foreign mature scales” (line 406) to “well-validated original English scales (e.g., Rifon et al., 2004; Assiouras et al., 2015),” which specifies the sources more clearly. In addition, we have replaced all “~” symbols with “–” in Table 1 as requested. We also reviewed Tables 2 and 3 and confirmed that no similar issues exist, ensuring consistency across all tables.

| **Table 1 .Sample Characteristics (n=322).** | | | | | | | |
| --- | --- | --- | --- | --- | --- | --- | --- |
| **Variable** | **Attribute** | **Frequency** | **％** | **Variable** | **Attribute** | **Frequency** | **%** |
| Gender | Male | 140 | 43.5 | Occupation | Student | 46 | 14.3 |
|  | Female | 182 | 56.5 |  | Enterprise and public institution | 85 | 26.4 |
| Age | 18-22 years | 28 | 8.7 |  | Professionals | 34 | 10.6 |
|  | 23-32 years | 169 | 52.5 |  | Agriculture, forestry, animal  husbandry and fishery | 11 | 3.4 |
|  | 33-42 years | 101 | 31.4 |  | Commercial and service  industry personnel | 120 | 37.3 |
|  | 43-52 years | 21 | 6.5 |  | Production and  transportation-related  personnel | 13 | 4.0 |
|  | 53 years and above | 3 | 0.9 |  | Self-employed | 5 | 1.6 |
| Education |  |  |  |  | Retiree | 3 | 0.9 |
|  | Junior high school | 6 | 1.9 |  | Others | 5 | 1.6 |
|  | Senior high school | 14 | 4.3 | Monthly income | Below 2,000 yuan | 27 | 8.4 |
|  | Junior college | 40 | 12.4 |  | 2000-2999 yuan | 17 | 5.3 |
|  | Bachelor | 238 | 73.9 |  | 3000-4999 yuan | 34 | 10.6 |
|  | Master | 22 | 6.8 |  | 5000-7999 yuan | 89 | 27.6 |
|  | Doctor | 2 | 0.6 |  | More than 8,000 yuan | 155 | 48.1 |

Thank you again for your meticulous guidance. Your attention to detail has helped us further improve the standardization and readability of the manuscript. If there are any similar details that need to be adjusted in the future, we will optimize them immediately!

**Question 8：**For CMV test, [line 464-466] sounds very quirky. I think you mean that you are comparing the baseline model with the model with Common Latent Factor (CLF) and if the model with CLF improves model fit substantially, then CMV may be problematic. Your writing is misleading and incorrect which treat CMV as a variable you toggle on/off.

**Response:** Thank you for your correction regarding the description of the CMV test. We agree that the original wording in lines 464–466 was misleading, as it could be interpreted as treating CMV as a variable to be toggled on and off. To address this, we have revised the section as follows. First, following recent research (e.g., Wu, Ye, & Li, 2024, Journal of Destination Marketing & Management), we note that the Harman’s single-factor test is widely accepted. Second, we have removed the inaccurate description of the Common Latent Factor (CLF) method and now rely solely on Harman’s single-factor test to assess CMV.

*Wu, M. Y., Ye, S., Ye, S., & Li, Q. (2024). How other customers influence customer citizenship behavior in theme parks: The role of customer-customer rapport. Journal of Destination Marketing & Management, 31, 100847.*

Thank you again for your meticulous guidance, which has helped us improve the scientific nature of the method description!

**Question 9：**[Line 472] “Cronbach’s coefficient” is incorrect as Cronbach’s alpha (α) is technically a coefficient of internal consistency. So “Cronbach’s alpha” or “Cronbach’s coefficient alpha” is the correct way. I suggest using the former for simplicity.

**Response:** Thank you for pointing out the issue with terminology. We have revised all instances of “Cronbach’s coefficient” to “Cronbach’s alpha” throughout the manuscript to ensure accuracy in statistical terminology.

**Question 10：**Regarding the practical implications, I don’t think [Line 711-717] are backed by the research findings. Since you don’t actually research into the customer journey, recommending strategies for different stages sound like you do. Please only recommend base on your findings and if possible, a clear link, despite brief, back to the research findings would make it easier to comprehend.

**Response:** Thank you for highlighting the issue in the practical implications section. We agree that the statements in lines 711–717 concerning the customer journey were not directly supported by our research findings. To address this, we have removed those statements and revised the section to ensure that all practical implications are clearly grounded in our empirical results. Specifically, the revised version now focuses only on how host–guest interactions influence customer tolerance behavior and motivational attributions, thereby maintaining a clear link between findings and implications.

At the service management level, cultivating authentic and emotionally rich host–guest interactions proves essential. Since altruistic attributions strengthen tolerance, managers should encourage hospitality practices perceived as genuine, such as attentive communication and sincere care. In contrast, mechanical or overtly promotional behaviors risk generating egoistic attributions, reducing tolerance. Managers should also recognize that extended stays magnify the effect of interpersonal interaction, making sustained relationship-building with long-term guests especially valuable.

The above-mentioned modifications have been highlighted in blue in the revised draft for your intuitive verification.

**-----------Minor comments**

**Question 1：**In term of the language, I recommend a thorough proof-reading or language editing. Many sentences can be improved to make the language much smoother. Below I provide some examples but not limited to [line 72-34, p.3] can be revised into "By examining these dynamics, this study seeks to advance theoretical understanding while offering practical insights to foster harmonious host–guest relationships and improve homestay management strategies."[line 633-634] can be revised into “These findings provide novel empirical support for applying attribution theory in future tourism and service research.”[line 113, p.4], "Theoretically, first, this research is ..." doesn't sound very naturally.

**Response:** Thank you for your careful review and suggestions on language improvement. We have incorporated all the specific examples you provided:

Page 3, lines 72–73: revised to “By examining these dynamics, this study seeks to advance theoretical understanding while offering practical insights to foster harmonious host–guest relationships and improve homestay management strategies.”

Lines 633–634: revised to “These findings provide novel empirical support for applying attribution theory in future tourism and service research.”

Page 4, line 113: revised to “This research makes several theoretical contributions. First, …”

In addition, the entire manuscript has undergone a thorough language edit by a professional with experience in publishing in English-language journals, to further improve grammar, word choice, and readability.

Acknowledgments

We extend gratitude to TopEdit (www,topeditsci.com) for its linguistic assistance during this manuscripts preparation.

**Question 2：**The discussion is in good shape. I further recommend indicating explicitly which one is for which hypothesis to make it much easier to follow through. For example, [Line 565] First, the research findings indicate…behavior (H1), etc. [Line 567], Chen et al. and Jeon and Shin are missing the years.

**Response:** Thank you for your positive evaluation of the Discussion section and for the two constructive suggestions. We have implemented both as follows:

Clear linkage to hypotheses: We have added explicit references to the corresponding hypotheses throughout the Discussion to improve readability. For example:

First, the findings confirm that interpersonal interaction significantly shapes customer tolerance behavior (H1).

Second, the analysis reveals that interpersonal interaction shapes customers’ attribution of motives, enhancing altruistic attributions while diminishing egoistic ones (H2a, H2b).

Third, customers’ motivational attributions directly shape their tolerance behavior (H3a, H3b).

Fourth, the results confirm that motivation attribution mediates the effect of interpersonal interaction on tolerance behavior (H4a, H4b).

Fifth, the results highlight the moderating effect of stay duration (H5a, H5b, H6).

Reference details: The missing publication years for Chen et al. and Jeon and Shin have been corrected. The sentences were revised to ensure accuracy and avoid repetition, for example:

This outcome echoes prior studies highlighting interpersonal interaction’s role in driving tourist behavioral outcomes. For instance, Chen et al. found that host-guest interaction affects tourists’ behavioral intentions and attitudes[85], while Lin et al. found positive effects on satisfaction and revisit intentions[20].

All changes have been marked in the revised manuscript for your review.

**Question 3：**Mismatch fonts observed, for example almost all Table’s title “Table 2…” and “Table 3…”, etc.

**Response:** Thank you for pointing out the inconsistency in table titles. We have carefully reviewed all table and figure titles (including Table 2 and Table 3) and standardized the formatting according to the journal’s style guidelines. These revisions have been marked in the revised manuscript for your review.

For clarity, the revised portions in the manuscript have been highlighted in blue. We are grateful for your continued support and welcome any further suggestions you may have for improving the quality of our work. Once again, thank you for your dedicated efforts and professional input.
